# Cortically-Controlled Population Stochastic Facilitation as a Plausible Substrate for Guiding Sensory Transfer across the Thalamic Gateway

**DOI:** 10.1371/journal.pcbi.1003401

**Published:** 2013-12-26

**Authors:** Sébastien Béhuret, Charlotte Deleuze, Leonel Gomez, Yves Frégnac, Thierry Bal

**Affiliations:** 1 Unité de Neurosciences, Information et Complexité (UNIC), CNRS UPR-3293, Gif-sur-Yvette, France; 2 Laboratorio de Neurociencias, Facultad de Ciencias, Universidad de la República Oriental del Uruguay, Montevideo, Uruguay; University of Pennsylvania, United States of America

## Abstract

The thalamus is the primary gateway that relays sensory information to the cerebral cortex. While a single recipient cortical cell receives the convergence of many principal relay cells of the thalamus, each thalamic cell in turn integrates a dense and distributed synaptic feedback from the cortex. During sensory processing, the influence of this functional loop remains largely ignored. Using dynamic-clamp techniques in thalamic slices *in vitro*, we combined theoretical and experimental approaches to implement a realistic hybrid retino-thalamo-cortical pathway mixing biological cells and simulated circuits. The synaptic bombardment of cortical origin was mimicked through the injection of a stochastic mixture of excitatory and inhibitory conductances, resulting in a gradable correlation level of afferent activity shared by thalamic cells. The study of the impact of the simulated cortical input on the global retinocortical signal transfer efficiency revealed a novel control mechanism resulting from the collective resonance of all thalamic relay neurons. We show here that the transfer efficiency of sensory input transmission depends on three key features: i) the number of thalamocortical cells involved in the many-to-one convergence from thalamus to cortex, ii) the statistics of the corticothalamic synaptic bombardment and iii) the level of correlation imposed between converging thalamic relay cells. In particular, our results demonstrate counterintuitively that the retinocortical signal transfer efficiency increases when the level of correlation across thalamic cells decreases. This suggests that the transfer efficiency of relay cells could be selectively amplified when they become simultaneously desynchronized by the cortical feedback. When applied to the intact brain, this network regulation mechanism could direct an attentional focus to specific thalamic subassemblies and select the appropriate input lines to the cortex according to the descending influence of cortically-defined “priors”.

## Author Summary

Most of the sensory information in the early visual system is relayed from the retina to the primary visual cortex through principal relay cells in the thalamus. While relay cells receive ∼7–16% of their synapses from retina, they integrate the synaptic barrage of a dense cortical feedback, which accounts for more than 60% of their total input. This feedback is thought to carry some form of “prior” resulting from the computation performed in cortical areas, which influences the response of relay cells, presumably by regulating the transfer of sensory information to cortical areas. Nevertheless, its statistical nature (input synchronization, excitation/inhibition ratio, etc.) and the cellular mechanisms gating thalamic transfer are largely ignored. Here we implemented hybrid circuits (biological and modeled cells) reproducing the main features of the thalamic gate and explored the functional impact of various statistics of the cortical input. We found that the regulation of sensory information is critically determined by the statistical coherence of the cortical synaptic bombardment associated with a stochastic facilitation process. We propose that this tuning mechanism could operate in the intact brain to selectively filter the sensory information reaching cortical areas according to attended features predesignated by the cortical feedback.

## Introduction

The thalamus is the major sensory gateway to the cerebral cortex. Forming the output of the retina, axons of ganglion cells diverge to connect a small number of thalamocortical (TC) neurons in the dorsolateral geniculate nucleus (dLGN); likewise several ganglion cells send convergent connections to individual TC neurons [1]. In turn, a sizable number of TC neurons (ranging from 15 to 125 in the cat [2]) converge onto individual recipient cortical neurons [3]. However, in spite of the fact that it is often described and modeled as a pure feedforward relay, the thalamus receives a massive corticofugal feedback. The functional interactions between the feedforward thalamocortical converging stream and the corticothalamic (CT) feedback are not known, and yet likely plays a key role in the control of the global gain and filtering features of the sensory thalamic relays.

Despite the fact that the function and mechanisms of the CT input have attracted much interest they are still a matter of discussion [4], [5]. A first accepted view is that the cortical feedback influences the transfer of sensory information by TC cells [6]–[8] and may participate to modulate visual responses during attention and awareness [9]. A second and well-publicized hypothesis endows the CT feedback and the thalamic nucleus reticularis (NRT) with a searchlight function [10] or focal attention [11] by enhancing selectively the receptivity of targeted TC neuron populations to attended sensory features. Others envision the thalamus as an “active blackboard” onto which the cortex could write down the results of its computation [12].

Nevertheless, the cellular mechanisms underlying the functional impact of the CT feedback are poorly understood despite a few experimental studies pointing to the spatial sharpening of thalamic receptive field and its ON-OFF antagonism [13], the facilitation of lateral geniculate nucleus (LGN) activity in the awake cat [14] or attentive monkey [15], the synchronizing action on thalamic neurons involved in the detection of co-aligned elements in the visual field [16], [17] or the enhancement of the surround antagonism during motion processing [18].

A more mechanistic view, which is the central working hypothesis of this paper, is that the cortex has the ability to gate the thalamic transfer of sensory inputs via “on-line modulation” of the transfer efficiency of TC neurons through the contextual synaptic bombardment originating from the CT input [19].

In the primary visual cortex (V1; areas 17 and 18 in the cat), layer 6 is the source of the CT synaptic feedback to thalamus. Activity patterns originating from projections of cortical layer 6 remain a major mystery although recent studies suggest behavioral circumstances in which the feedback from corticothalamic neurons could be engaged [20]. In the mouse, layer 6 neurons projecting to thalamus are spontaneously active and their activity increases during unspecific full-field visual stimulation (Fig. 1D in [21]). However, the fine-scale activity of layer 6 circuits during naturalistic vision is unknown. A detailed modeling of the activity of layer 6 seems presently an unreachable target, since it would require including interrelations with all other cortical layers and other related cortical areas (see [20], [22]). Instead, our strategy has been to model the top-down cortical input as a configurable activity pattern transmitted by excitatory and inhibitory synapses for which we have fully explored the parameter space.

**Figure 1:**
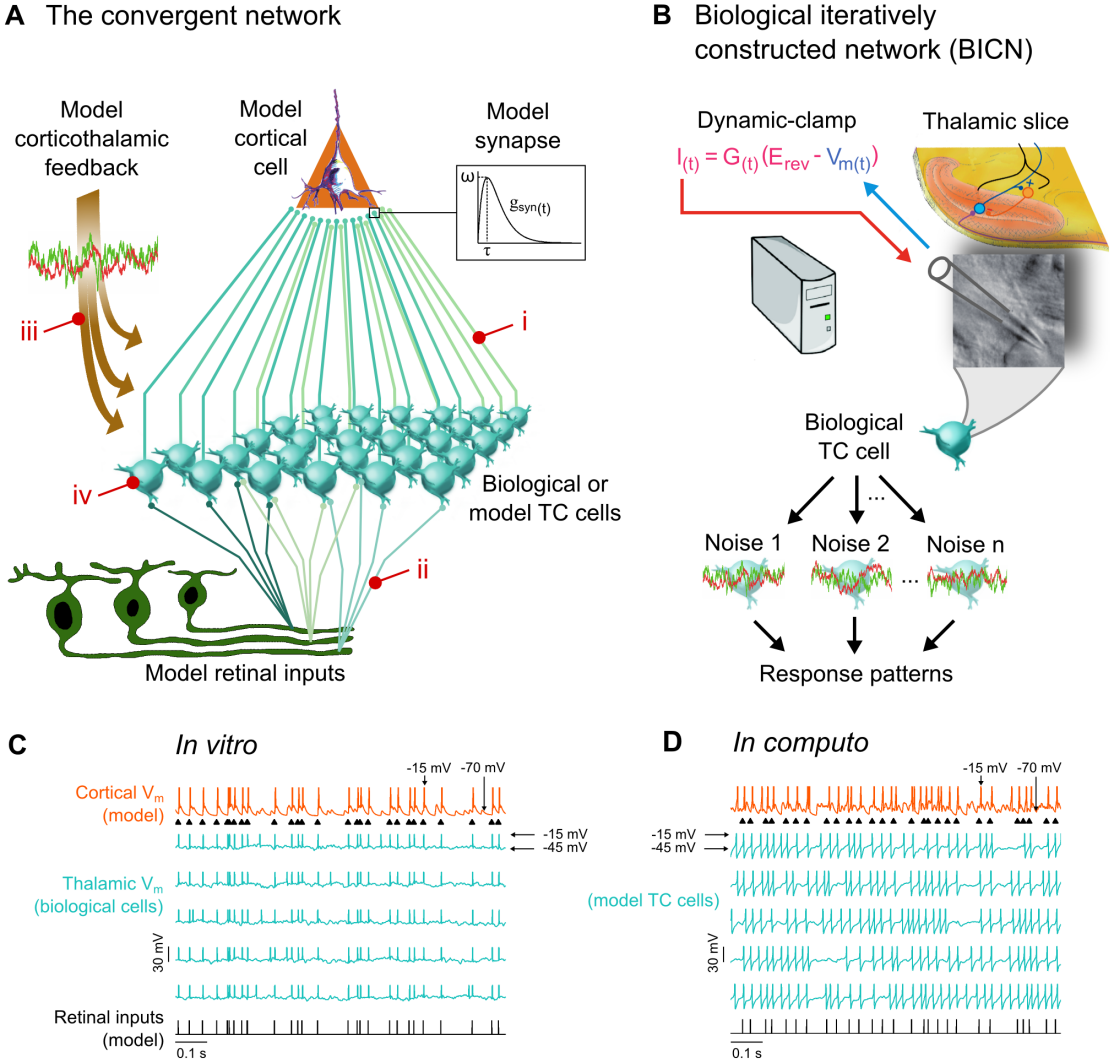
*In vitro* and *In computo* reconstructions of convergent thalamocortical networks. A. Hybrid convergent circuit model. Biological or model TC cells synaptically converge to a model cortical neuron. The population of TC cells is fed with modeled retinal inputs and receives a corticothalamic input mimicked through the injection of stochastically fluctuating mixed excitatory and inhibitory conductances. Inset. Retinal and thalamic synaptic inputs elicit somatic conductance-based events in the target neurons (see Methods). i, ii, iii, iv. Critical parameters of the circuit explored in this study. See text for more details. B. BICNs consisted in at least one biological TC cell recorded multiple times with identical retinal inputs and varying patterns of corticothalamic synaptic noise injected in real time through dynamic-clamp. The obtained response patterns were then simultaneously replayed in the hybrid circuit thus mimicking the functional impact produced by thalamocortical convergence. C. Membrane potential traces for a BICN. A single TC cell was recorded sequentially using the same model retinal inputs but adding different realizations of a synaptic stochastic bombardment each sharing the same conductance mean and variance (see Methods; this is the uncorrelated condition in Fig. 4 and 5). Only five out of the ten thalamic voltage traces are shown. Spikes were truncated to −15 mV. D. Same as C with model TC cells.

At the single-cell level, the efficiency of the transfer via the thalamus was established by measuring the spiking probability function of individual TC relay cells, and shown to depend on the statistical context of the synaptic bombardment [19]. When considering the whole population level, the question we want to address is whether cortically-induced modulation of the thalamic transfer efficiency can be deduced solely from the interactions observed at the single-cell level or if it emerges from higher order interactions within the network. In other words, in terms of the global information transfer between retina and cortex, is the combined effect of changes operating in individual cells equivalent to the modulation of the thalamic population as a whole? Our working hypothesis is that the CT synaptic bombardment is able to modulate the transfer efficiency of specific TC neurons, not only at the single-cell level by impacting on the input-output gain [19], but also at the population-level by controlling the contextual correlations in membrane potential fluctuations within subgroups of TC relay cells. We present here a new approach in the study of the sensory transfer gating mechanisms in the thalamus by exploring the functional impact of higher order interactions arising between multiple TC neurons, both in computer models and in the slice.

## Results

The aim of our experimental plan was to combine *in computo* models of the retino-thalamo-cortical (RTC) pathway and top-down corticothalamic inputs with *in vitro* measures of information transfer at different points of the circuit. The results are organized consequently to describe the global circuit model and its various implementations, present parametric studies of the dependency on the model on various structural and activity-dependent features, and quantify their functional impact on global information transfer efficiency between retina and cortex.

More specifically, the first part of the results and the methods present the implementation of the circuit model (Fig. 1A) and biological iteratively constructed networks (BICNs) *in vitro* (Fig. 1B). In the second and third parts, respectively, we tested critical structural parameters of the thalamocortical and retinothalamic circuits topologies (Fig. 1A, i and ii). The fourth part shows the dependency of the model behavior on CT synaptic bombardment statistics (Fig. 1A, iii). In the final parts, we implemented various contextual patterns in the thalamic layer, including membrane potential fluctuation correlation across TC cells imposed via the CT input, in both topologically optimized BICNs and model networks (Fig. 1A, iv).

In all simulations, mutual information analysis (Eq. 19) was carried out to estimate the efficiency of the global information transfer between the retinal input and the cortical response (later referred as “transfer efficiency” (TE); see Methods) [23], [24]. This theoretical tool quantifies the non-linear statistical dependencies of specific features between two spike trains such as spike events, absence of spikes or any combinations of these two events in a given time window (see Figure S1 for comparison with other methods).

### The thalamocortical convergence circuit model

In our model, the topology of the feedforward retino-thalamo-cortical circuitry (Fig. 1A) is highly schematic, but constrained with detailed biophysical measurements taken from the available literature. It is composed by an ordered layout of populations of thalamocortical neurons in the dLGN converging to a single layer 4 pyramidal neuron of the primary visual cortex (see Methods for details). Circuits were either built from collections of Hodgkin Huxley type model neurons (Eq. 1–3) or reproduced in an *in vitro* slice preparation of the rat thalamus using an iterative procedure [25] implemented in dynamic-clamp [26]–[28]. Synapses were conductance-based (Fig. 1, inset; see Methods) and mimicked AMPA (α-amino-3-hydroxy-5-methyl-4-isoxazolepropionic acid) and GABA_A_ (gamma-aminobutyric acid type A) mediated current flows (Eq. 5 and 6). We based our circuit reconstructions on direct estimates of the structure and size of the elementary thalamic microcircuitry found in the literature. The topology of the circuit was parametrized to test the sensitivity of information transfer on the structural constraints. We varied in the model simulations (Fig. 1A, i) the degree of convergence and weight of TC synapses onto a single target cortical neuron and (Fig. 1A, ii) the divergence/convergence configuration of the retinogeniculate axons and retinal input synchronization.

In order to reproduce the main components of the thalamic input, each artificial or biological TC neuron was fed with an artificial retinal input pattern (Eq. 10) and received cortical inputs simulated by stochastically fluctuating conductances composed of mixed excitatory and inhibitory inputs (see Eq. 7 and 8). Exploring the effects of the CT inputs requires modeling an artificial synaptic signal whose statistical signature can be experimentally controlled. With this approach it is possible to generate a large range of artificial CT activity patterns and explore their effects by stimulating recipient neurons with the resulting contextual synaptic bombardments. It should be emphasized that we simulate here the contextual synaptic noise in an open-loop fashion. In other words, it does not depend on the activity of the model cortical cell but rather is controlled by a set of statistical parameters. This contextual synaptic noise produced background membrane voltage fluctuations in TC neurons and was designed such as to mimic the dynamics of the direct AMPA synapses and the disynaptic GABAergic input originating from local interneurons and neurons from the NRT in the wake state where the cortical input is presumably irregular. A distinctive feature of this paradigm is that each one of the TC neurons can be either modulated by a neuron-specific pattern of synaptic fluctuations or share common synaptic inputs with neighboring cells. In order to control the functional impact of the CT input, we analyzed critical factors of the statistics of this synaptic noise such as the ratio of inhibitory versus excitatory conductances, the amplitude of the conductance fluctuations and the level of coherence of the noise shared by TC neurons. Relevant model parameters are summarized in Table 1.

**Table 1:** Retino-thalamo-cortical model circuit parameters.

Parameter	Description	Value(s) or range
Retinal stimulation		
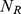	Number of retinal cells	1, 15 cells
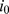	Mean interspike interval (firing rate)	0.33 ms (30 Hz)
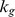	Shape parameter of the gamma distribution	3
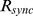 (1)	Retinal input synchronization	0–1
Thalamocortical cells		
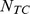	Number of TC cells	1–240 cells
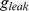	Leak/passive conductance	9.12 nS
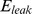	Leak reversal potential	−76.5 mV
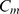	Membrane capacitance	0.21 nF
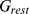	Resting input conductance	8.34 nS
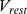	Resting membrane potential	−74.3 mV
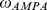	AMPA synaptic weight	12.5 nS
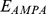	AMPA reversal potential	0 mV
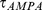	AMPA time to peak amplitude	1 ms
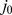 (2)	Spike-time mean jitter	0–10 ms
Cortical cell		
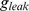	Leak/passive conductance	29.0 nS
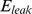	Leak reversal potential	−70.0 mV
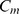	Membrane capacitance	0.29 nF
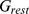	Resting input conductance	33.4 nS
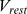	Resting membrane potential	−70.6 mV
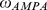	AMPA synaptic weight	0–40.0 nS
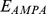	AMPA reversal potential	0 mV
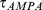	AMPA time to peak amplitude	1 ms
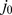 (2)	Spike-time mean jitter	0–10 ms
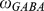 (3)	GABA synaptic weight	0–10.0 nS
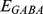 (3)	GABA reversal potential	−75 mV
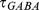 (3)	GABA time to peak amplitude	2 ms
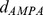 (3)	GABA input time lag (relative to AMPA)	0–10 ms
Synaptic bombardment		
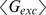 ( 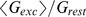 )	Excitatory conductance mean (amplitude)	0–25.02 nS (0–3)
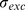 ( 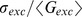 )	Excitatory conductance SD (variation ratio)	0–12.51 nS (0–1)
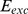	Excitatory conductance reversal potential	0 mV
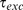	Excitatory conductance time constant	2.7 ms
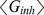 ( 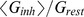 )	Inhibitory conductance mean (amplitude)	0–25.02 nS (0–3)
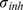 ( 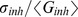 )	Inhibitory conductance SD (variation ratio)	0–8.34 nS (0–1)
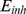	Inhibitory conductance reversal potential	−75 mV
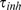	Inhibitory conductance time constant	10.5 ms
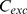 (4)	Exc./Inh. conductances correlation	0–1
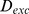 (4)	Inh. conductance time lag (relative to Exc.)	0–10 ms
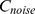 (5)	Synaptic noise correlation	0–1

^(1)^ Retinal synchronization (implemented for 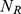 = 15).

^(2)^ Presynaptic inputs random time jitters.

^(3)^ Feedforward inhibition in the cortical cell.

^(4)^ Temporal correlation of excitatory and inhibitory inputs in single TC cells.

^(5)^ Temporal correlation of synaptic inputs across TC cells.

We used BICNs *in vitro* and computer modeling (Fig. 1B) to reproduce and explore systematically the voltage dynamics of neural circuit existing in the intact brain. The activity patterns of biologically recorded relay TC cells were replayed to simulate the synaptic convergence activity of the thalamic layer and stimulate the modeled cortical cell in the primary visual cortex, similar to that described above in the model circuit. A BICN hybrid thalamic layer thus consisted of a population of a parametrized set of pseudo-neurons, whose output trains replayed simultaneously individual response patterns recorded sequentially in biological TC neurons (see Methods).

Figures 1C and 1D show examples of voltage traces of TC neurons chosen among a larger population for both BICNs (*in vitro*) and model (*in computo*) circuits. The fluctuating voltage recordings illustrated for the TC cells are the result of different synaptic bombardment sequences for each trace. The synaptic bombardment has been optimized in order to maximize the transfer efficiency according to paradigms explored in later sections.

### Parametric dependency on thalamocortical convergence and synaptic weight

In this first set of simulations, the statistics of the corticothalamic input were uniform across the whole thalamic population but the individual time patterns were chosen to be independent between each of the TC cells. The entire thalamic population was connected by a unique retinal cell mimicking the discharge pattern of an ON-center Y cell (30 Hz gamma 3 distribution, [29], [30]; see Eq. 10). Hence, the TC cells input differed only in their individual corticothalamic synaptic noise pattern.

**Figure 2:**
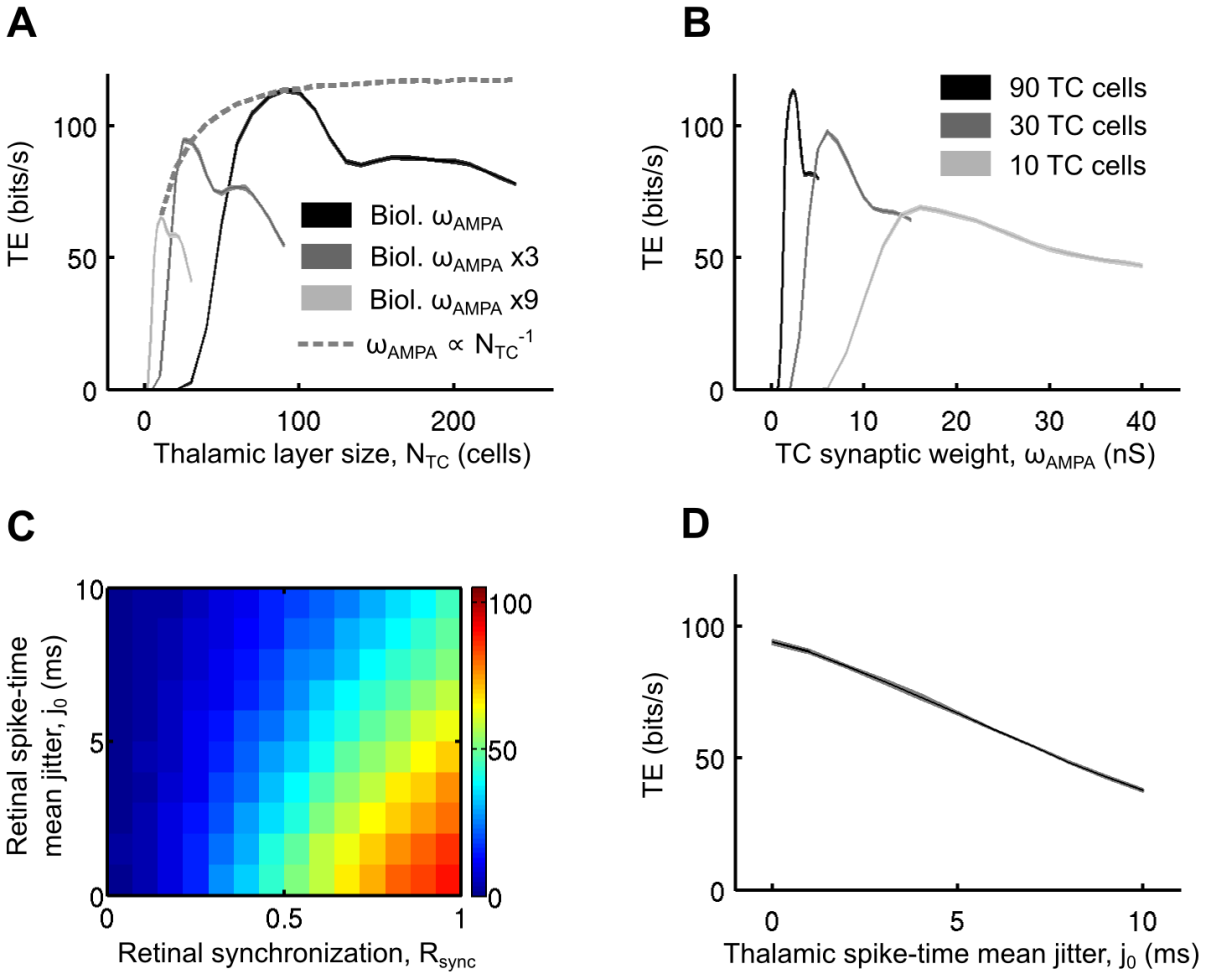
Network topology affects the retinocortical transfer of sensory information *in computo*. A. Transfer efficiency as a function of the thalamic population size. Each point represents the simulation of a modeled convergent circuit for three predefined TC AMPA synaptic weights in addition to a special case (dashed line) where the synaptic weight was adjusted to the thalamic population size on a per-simulation basis (see text for more details). The thickness of the curves represent the standard deviation across ten repetitions of the same retinal sensory simulation realized each time in the context of a different realization of the cortical synaptic bombardment. B. Transfer efficiency as a function of the TC AMPA synaptic weight for three predefined thalamic population size. C. Influence of the level of retinal input synchronization. The TE was measured for varying retinothalamic spike-time mean jitters and retinal synchronization levels (see text for more details). D. Transfer efficiency measured as a function of the thalamocortical spike-time mean jitter.

We adjusted the synaptic weight (see Eq. 6 and inset in Fig. 1) of the thalamocortical synapse to a biologically realistic value (∼2.3 nS, [31]) and varied the population size (Fig. 2A, black curve). The TE was shown to reach a maximum for a convergence ratio of 80–100 cells. In addition, we observed in this configuration that roughly the simultaneous firing of a third of the TC cells was required to elicit a spike in the cortical neuron. These ballpark estimates were justified by protocols using single AMPA events in which a strength of ∼80 nS was required to evoke reliably a cortical spike, corresponding to 30–35 TC neurons firing simultaneously (Fig. S2A). Smaller thalamic population sizes resulted in an insufficient synaptic drive of the target cortical cell while larger thalamic population sizes led to an increased amount of cortical spikes decoupled from the retinal input, both of these cases leading to inefficient transfer values.

We then froze the thalamic population size to 90 cells and varied the thalamocortical AMPA synaptic weight (Fig. 2B, black curve). The TE peak was obtained for a biologically realistic synaptic weight (2–2.5 nS) thus confirming the value chosen in Figure 2A.

The above 90 cells version of the model circuit is too large to achieve successful biological thalamic layer reconstruction and is computationally intensive for multi-dimensional parametric explorations. We therefore reduced the number of TC relay neurons in the model circuit and ran simulations to find the optimal synaptic weight reflecting this decrease. First, we designed a 30 cells model circuit that was used in later computational explorations (Fig. 2A and 2B, dark-gray curves). Second, we tested a 10 cells model circuit as a control to match the BICN hybrid thalamic layer size presented in Figure 1C and developed in later sections (Fig. 2A and 2B, light-gray curves). When reducing the population size from 90 to 30 TC cells, a corresponding increase of the synaptic weight by a factor of 3 was necessary and sufficient to maintain optimality in signal transfer. The same compensatory rule held when lowering the population size from 30 to 10 TC cells. Hence, topologically optimized networks consisted of 90 TC cells with the biologically realistic synaptic weight, 30 TC cells with a 3 fold increase of the weight or 10 TC cells with a 9 fold increase of the weight.

We tested other optimized topologies according to the following empirical rule which reflects the above findings: “number of TC cells”×“TC synaptic weight”≈210 nS (Fig. 2A, dashed curves). This empirical rule ensured that the total net summed synaptic input received by the target cortical cell was constant thus enabling us to isolate the effect of the population size parameter. An asymptotic saturation behavior was observed, showing a ceiling value in the TE for convergence ratios around a critical value of 90 TC neurons. This finding implies that the structure of the convergent networks, albeit flexible, needs to be constrained in order to provide an efficient and optimal information transfer.

In the subsequent investigations and unless mentioned otherwise, we performed numerical investigations with models of parallel feedforward lines composed of 30 TC relay cells converging to one model cortical cell through TC synapses using a weight optimized as described above (Fig. 2B, dark-gray curve, 7 nS).

### Parametric dependency on the synchronization level of retinal inputs and TC spikes

Multiple retinal input lines were added to the model circuit described previously. Both convergent and divergent processes have been documented between retinal ganglion cells and relay thalamocortical cells in the LGN [1]. The 30 TC relay cells were contacted by 15 retinal cells in a realistic mixture of divergent and convergent processes as illustrated in Figure 1A. Each retinal cell contacted 4 TC neurons and each TC neuron was contacted by 2 retinal cells [1]. The thalamic population size and the TC synaptic weight were kept frozen. The cortical synaptic bombardment was kept as described above.

The level of synchronization of the retinal afferents was controlled in two ways. First, we varied the number of retinal cells replaying an independent activity pattern resulting in graded levels of synchronization controlled by the parameter 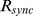 (Eq. 11). Second, we introduced an ad-hoc jitter to randomly shift the timing of each retinal spikes (see Methods). The average spike-time shift was characterized by the mean jitter parameter 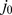 (Eq. 12). Low 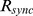 values and large spike-time jitters (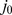) led to desynchronized retinal inputs in the TC relay cell population. In this model circuit including more than one retinal cell, transfer efficiency was measured between only one of the retinal cells and the cortical response. The chosen reference retinal cell was always the one whose activity was correlated with some or all of the retinal cells in the synchronized retinal input conditions.

Results show, as expected, that the transfer efficiency of the model circuits increased with the retinal synchronization (Fig. 2C, x-axis). Similarly, the TE value dropped for large spike-timing jitters but remained robust with low jitters (Fig. 2C, y-axis) with a decrease of less than 20% for jitters up to 3 ms. We applied a similar paradigm to the thalamic spikes and found a very similar result. The TE scaled nearly linearly with the thalamic spike-time jitters and remained robust with low jitters (Fig. 2D).

Because biological-like retinothalamic lines with highly synchronized retinal inputs behave like divergent networks made of a single retinal ganglion cell contacting the entire thalamic population, we used this later paradigm for the rest of the exploration.

### Parametric dependency on the statistics of the contextual synaptic bombardment

These simulations used optimized networks consisting of 30 TC cells fed by a single retinal cell. Parameters controlling the topology of the model circuits, such as the thalamic population size and the TC synaptic weight were kept constant. We explored various statistical configurations of the cortical input so that each TC cell received a unique realization of a synaptic bombardment while the global statistics seen by each cell remained identical, thus corresponding to an uncorrelated bombardment among the dLGN population similar to what was done in previous sections (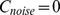, see Methods).

**Figure 3:**
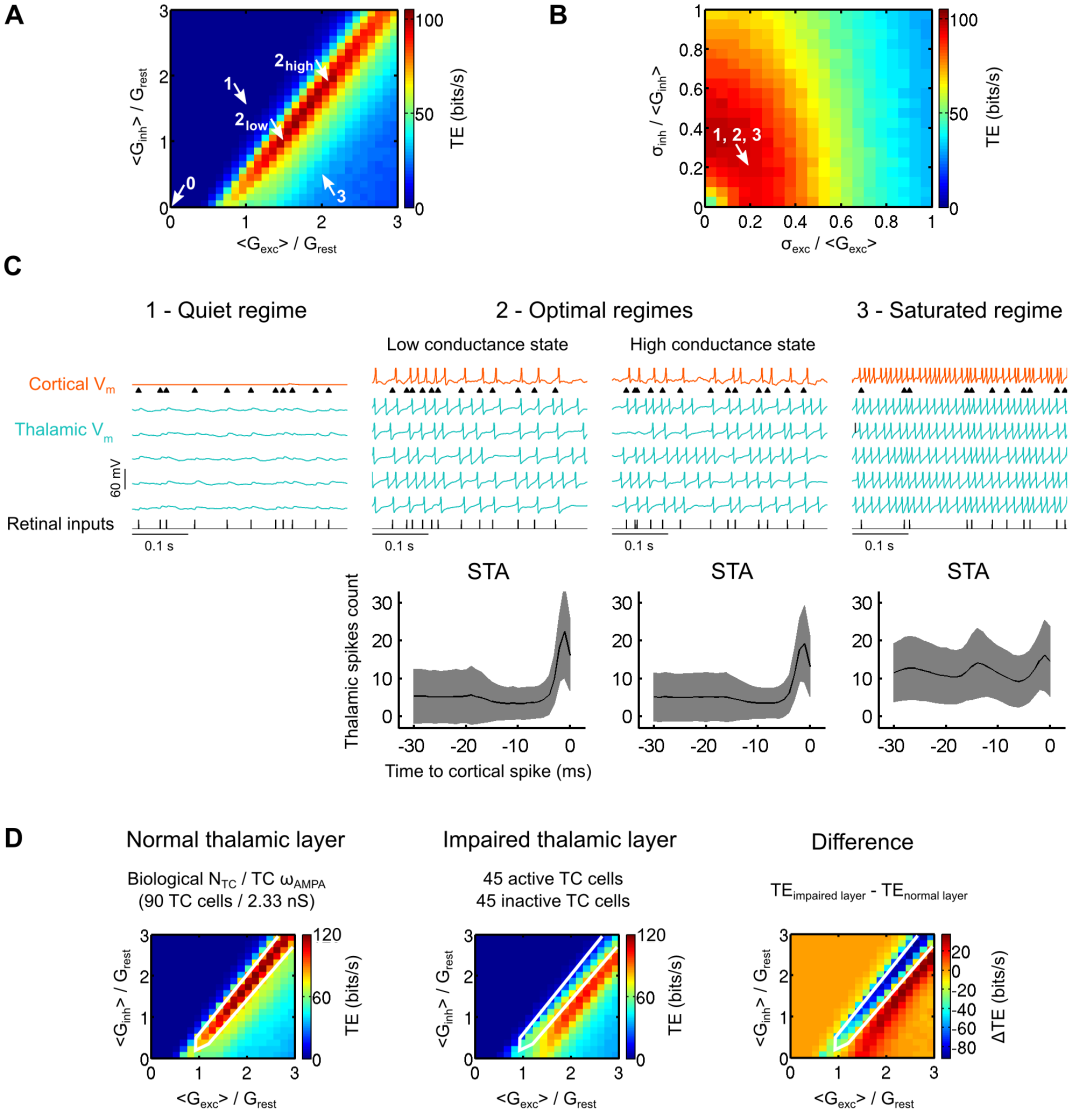
Background synaptic bombardment tunes the retinocortical signal transfer *in computo*. A. Effect of the cortical input mean excitatory and inhibitory conductances on TE. Cortical input conductances are normalized relative to the rest conductance of the TC cells. For each trial, each model TC cell in the circuit received a unique realization of the synaptic noise conductances, obeying the same statistics across trials (this condition is referred as the uncorrelated condition in later figures). Arrows denote specific operating regimes, which are shown in C (arrows 1 to 3) and in Figure S3A (arrow 0). The conductance variation ratio was fixed to 0.2 for both the excitatory and inhibitory components, an optimal value denoted by the arrow in B. B. Similar to A for the SD of the conductances. The SD of the conductances were normalized relative to their respective means. The conductance amplitudes were set to 1.5 and 1.0 for the excitatory and inhibitory components of the synaptic noise, respectively. These optimal values are denoted by the arrow “2_low_” in A. C. Top. Membrane voltage traces for three operating regimes reflecting three distinct cortical synaptic bombardment statistics. Each regime is shown by an arrow in A and B. The optimal regime was further separated into a low and a high conductance state. Bottom. Cortical spike-triggered averages relative to the number of thalamic spikes were calculated for each of the above cortical voltage traces. The number of thalamic spikes preceding each cortical spike was averaged and plotted as the black curve. Grayed areas indicate the SD of the thalamic spikes count across all cortical spikes. D. Numerical explorations as in A for a control circuit of normal biological size, and for an impaired circuit in which half of the thalamic cells were inactive. The TE difference was calculated for each point by substracting the TE obtained for the normal layer from the TE obtained for the impaired layer. White lines delineate the red ridge of optimal transfer found in the control condition, which is replicated in the two other graphs for comparison.

We varied the mean and standard deviation (SD) of both excitatory and inhibitory components of the synaptic bombardment (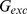 and 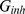, respectively) such as to maximize the efficiency of the information transfer within the model network. Mean synaptic conductances were normalized relative to the rest conductance of the TC neurons (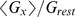 or “conductance amplitude”; x substitutes to “exc” or “inh”) and SD were normalized relative to their respective mean (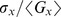 or “conductance variation ratio”) (see Methods). The rest conductance, 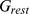, defines the input conductance of the cells at their resting potential and is approximately equal to the leakage passive conductance, 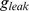, when measured in absence of external input activity. First, we ran coarse four-dimensional explorations of the mean and SD for both the excitatory and inhibitory components of the synaptic bombardment. Finer explorations were then narrowed around the optimal estimates by keeping constant either the standard deviation (Fig. 3A) or the mean conductances (Fig. 3B).

In Figure 3A the conductance variation ratio was fixed at 0.2 for both the excitatory and inhibitory components (an optimal value chosen in Fig. 3B). An exhaustive exploration of the conductance parameter space revealed the emergence of a ridge (dark red) within a narrow band, where the TE is highest for an ensemble of pairs of excitatory and inhibitory conductance amplitudes. This indicates that an adjustment in the balance between excitation and inhibition is required to optimize the information transfer.

On the left side of the narrow band (arrow “1” corresponding to the “quiet” regime domain shown in Fig. 3C; 0 bit/s), information transfer is inefficient due to the concomitant action of strong inhibition and weak excitation, resulting in an effective silencing of the TC cells.

The normalized total cortical input conductance (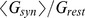; see Eq. 9) is a convenient way to characterize the relative strength of the cortical input action on the TC cells. In the band delineating optimal information transfer, the TE is highest for normalized total cortical input conductance ranging from ∼2.5 to ∼4. Two optimal background conductance states connected by the ridge of optimal TE values can be qualitatively distinguished. The first state is a low conductance (LC) regime (denoted by the arrow “2_low_” in Fig. 3A; 95 bits/s for 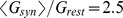) where the mean values of excitatory and inhibitory conductances are approximately comparable to the rest conductance. The second state is a high conductance (HC) regime (denoted by the arrow “2_high_” in Fig. 3A; 97 bits/s for 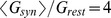), characterized by a rest conductance that is approximately 50% smaller than the mean values of excitatory and inhibitory conductances. In the corresponding regimes of activity (LC and HC optimal regimes shown in Fig. 3C), the cortical spike-triggered average (STA) clearly indicates an increase of the thalamic synchrony a few milliseconds before the cortical spikes. No major differences were observed apart from slightly stronger voltage fluctuations in the relay cells for the HC state (TC cells membrane potentials SD after removal of spikes is 1.0 mV for LC and 1.4 mV for HC) and a slightly sharper peak for STA in the LC state. No significant STA was presented for the quiet regime since no cortical spikes were evoked.

On the right side of the narrow band (arrow “3”, corresponding to the “saturated” regime domain shown in Fig. 3C; 31 bits/s), the inefficiency of the transfer is provoked conversely by a saturating level of excitation. The resulting spiking regime in the relay cells was sufficient to excite the cortical cell in a tonic mode and decorrelate its spiking from the timing of the retinal input, as shown in Figure 3C by the cortical spike-triggered average.

Next, we kept constant the excitatory and inhibitory conductance amplitudes (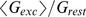 = 1.5 and 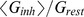 = 1.0 as found to be optimal in Figure 3A and corresponding to the LC state in Fig. 3C) and varied the conductance variation ratio (Fig. 3B). A ring shaped area of optimal transfer was found (arrow in Fig. 3B), flanked by areas where both either very low or very high fluctuations led to an inefficient transfer. Note that the amount of inhibitory fluctuations had limited importance compared to the amount of excitatory fluctuations as shown by the enlargement of the ring over the y-axis. One explanation resides in the fact that the inhibitory reversal potential is close to the actual resting potential of the model TC cells, effectively limiting the amplitude changes of the inhibitory synaptic bombardment fluctuations, and thus their effect on stochastic resonance (see below).

**Figure 4:**
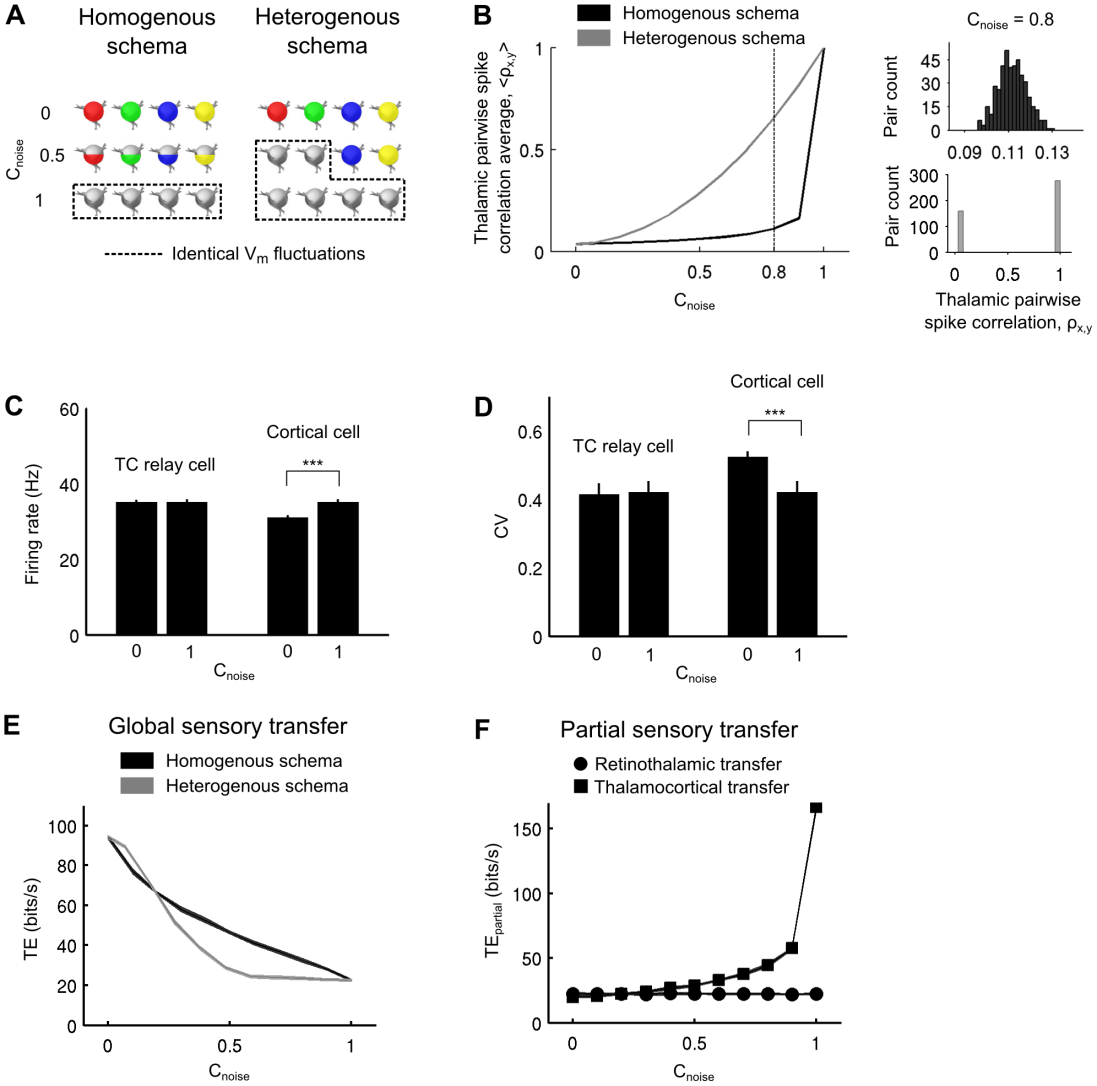
Impact of the CT inputs correlations on retinocortical information transfer efficiency *in computo*. A. Illustration of the two synaptic bombardment correlation schemas used in this study. Colored cells receive an identical synaptic noise. Non-colored cells received an independent synaptic noise. Partially colored cells receive a partially correlated synaptic noise. See text for more details. B. Left. Mean pairwise spike correlations among the whole thalamic population as a function of the synaptic noise correlation strength, 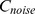. Right. Example distributions of the thalamic pairwise correlations for 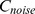 = 0.8 (indicated by the vertical dashed line in the left graph) for the homogeneous (upper) and heterogeneous (lower) schemas. C. Thalamic mean firing rate (± SD across the whole thalamic population) and cortical firing rate (± SD across non-overlapping windows of the cortical spike train). D. Thalamic coefficient of variation (± SD across the whole thalamic population) and cortical coefficient of variation (± SD across non-overlapping windows of the cortical spike train). E. Effect of the synaptic bombardment correlation strength on TE for both correlation schemas illustrated in A. F. Retinothalamic and thalamocortical partial sensory transfer efficiencies (TE_partial_) for the homogeneous correlation schema. See text for more details.

The mean firing rate of the TC cells occurring under optimal synaptic bombardment (35 Hz) was slightly higher than both the retinal and cortical firing rates (30 Hz) (Fig. 4C, 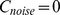). The additional spikes responsible for the increased thalamic firing were caused by the CT input as expected from the high probability (0.7) to evoke a spike under optimal synaptic bombardment, even for retinothalamic AMPA events of small amplitude (Fig. S2B, gray curve). In contrast, in absence of contextual synaptic bombardment (denoted by the arrow “0” in Fig. 3A where 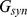 = 0; 1 bit/s; traces shown on Fig. S3A), thalamic spikes were solely evoked by the retinal inputs with a much lower probability (Fig. S2B, black curve) and TC cells relayed significantly fewer spikes than present in their retinal inputs. Depolarizing the thalamic cells with a positive constant current (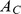, Eq. 13), as to mimic the effects of neuromodulation (see Discussion), shifted the optimal response ridge seen in Figure 3A towards lower 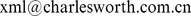 values, and increased the baseline TE observed in absence of synaptic bombardment (Fig. S3B; 50 bits/s for 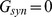 and a 0.6 nA constant current).

A common feature in the thalamocortical circuit is feedforward inhibition (FFI). FFI is defined here in a loose sense (not cell specific). It consists of a group of TC cells that influences excitatory cortical cells in layer 4 through direct connections and indirectly through local relay inhibitory neurons. In such FFI circuits, postsynaptic excitatory neurons are considered highly sensitive to the relative timing of action potentials among presynaptic TC neurons (reviewed in [32]). Therefore, we tested the impact of FFI in the current model circuit (see Methods). In the cortical cell, inhibitory GABA_A_ disynaptic events triggered by thalamocortical inputs closely followed direct excitatory AMPA monosynaptic events. The FFI was controlled by the GABA_A_ synaptic weight and its time lag relative to the AMPA events. We numerically explored a range of GABA_A_ synaptic weights and time lags and found that the sensory signal transfer efficiency was improved in the saturated regime by an average of more than 50% for GABA_A_ synaptic weights ranging from 3 to 5 nS and time lags up to 3 ms (Fig. S4B; note this range of synaptic weights corresponds to the combined inhibitory synaptic weight for the cortical cell in the 30 TC cells version of the model circuit). In contrast, TE in the optimal regimes was mostly unaffected for a large range of biologically realistic parameters, with only minor improvements characterized by a TE increased up to 3% and 5% for the LC state and the HC state regimes, respectively (Fig. S4A).

In the following sections, numerical simulations were performed using the optimal LC state of the synaptic bombardment. No FFI was implemented in the subsequent circuits.

### Contextual synaptic bombardment adaptation to impaired topology

We investigated if the drop in transfer efficiency observed when changing the size of the thalamic population without readjusting the TC synaptic weight (Fig. 2B, solid curves) could be counter-balanced by a different tuning of the cortical input. This question explores a potentially important issue, since it aims at determining if pathological impairments of sensory afferent circuits associated with degenerative diseases such as age-related macular degeneration, phantom limbs, tinnitus or strokes (see Discussion), could be compensated by corticofugal activity adaptation. We compared numerical explorations of the mean conductance amplitudes of the synaptic bombardment as was done in Figure 3A for an optimized network of realistic biological size (Fig. 3D, normal thalamic layer including 90 TC cells) and an impaired network where half of the TC cells did not receive any input (Fig. 3D, impaired thalamic layer including 45 active TC cells and 45 inactive TC cells). The TC synaptic weight of both normal and impaired thalamic layers are identical and set to the biological value which is optimized for a total of 90 TC cells.

As shown by the TE difference calculated by subtracting the normal TE from the impaired TE (Fig. 3D, TE difference), the transfer found in the control optimal narrow band for the normal thalamic layer (delimited by white lines) was degraded in the impaired thalamic layer, resulting in a large drop of transfer efficiency (decrease up to 102 bits/s in the control band). However, recovery was partially obtained in the impaired thalamic layer via a moderate shift of the optimal transfer ridge. The recovered peak efficiency (94 bits/s) accounted for more than 75% of the original peak transfer efficiency in the normal thalamic layer (121 bits/s). The recovered optimal band was slightly shifted towards higher 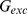 values indicating that it is possible to compensate for a decrease in the feedforward retinothalamic synaptic transmission by boosting the responsiveness of the TC cells through cortical synaptic bombardment.

We speculate that this compensation could occur in early stages of macular degeneration, but it would not work in later stages when the thalamic layer is too massively impaired by a drastically reduced number of retinal inputs. In our model, heavy compensation involved a large increase of the excitatory component of the CT input and drove the thalamocortical system into the saturated regime shown in Figure 3C, where the output of the thalamic population remained independent from the retinal afferent drive.

### Impact of the temporal coherence of the corticothalamic input across TC cells

Temporal correlations of corticothalamic inputs can be examined at two different scales in the thalamic layer, at the level of single cells or at the level of the cell population, with two very different outcomes for information transfer.

At the single cell level, the corticothalamic input directly excites TC cells and indirectly inhibits them via the NRT and local interneurons in the LGN [33]. The precise spatial and temporal organization of these inputs is not known. Inputs from layer 6 to TC and NRT cells may overlap if they originate from the same cortical columns [34], or not if they originate from different columns [13]. Therefore it can be hypothetized that different degrees of temporal correlation occurs in the target TC cells, between monosynaptic cortical feedback excitatory postsynaptic potentials (EPSPs) and disynaptic inhibitory postsynaptic potentials (IPSPs). To explore this question, we tested for a large range of correlation strengths (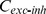) and correlation time lags (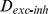) between the excitatory and the inhibitory components of the synaptic bombardment (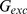 and 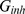, respectively; see Eq. 15 and 16). Positive correlation time lags caused the inhibition to lag behind the excitation. We observed only a very small decrease in the retinocortical transfer efficiency for high correlation level with no clear dependencies on the time lag (Fig. S5).

So far, the effects observed at the cell level can be explained by a classical gain control where the spike response probability of each individual TC cell is shaped by the characteristics of the noise bombardment [13], [19], [35].

Beyond this modulatory effect specific of each cell, the following simulations unravel another feature, critical in the control of information transfer, namely the temporal coherence of the synaptic bombardment across TC cells. To illustrate the functional impact of temporal coherence, two possible correlation schemas were explored at the population level, allowing a parametric exploration ranging from complete desynchronization (case examined so far in the previous parts) to full synchronization of the CT input across the whole thalamic cell population (Fig. 4A).

In the “homogeneous” correlation case, the cortical projections were arbitrarily divided into two sets of additive noise sources, correlated and uncorrelated, whose relative influence could be titrated parametrically: i) a pool of “shared” CT axons was distributed jointly to all cells of the population and provided a common synaptic input, leading to cross-cell correlations, whereas ii) a pool of “independent” CT axons targeting different cells was distributed within the population, thus providing desynchronized synaptic drive. The differential recruitment of these two types of projections by the cortex can be seen as a simple way to impose different amounts of correlation across the thalamic cells.

We also explored a “heterogeneous” correlation case, where only one type of input could be integrated at once by the thalamic cells which received either shared or independent CT inputs from the cortex. In this latter case, gradual correlation levels are just implemented by spatial heterogeneity in the recruitment by CT shared axons, where a variable number of TC cells receive shared CT inputs while the remaining cells receive independent CT inputs. This spatially organized correlation schema is illustrated by islands of neighboring thalamic cells being densely contacted by common CT axons which would be either synchronously activated by the cortex or kept inactive.

Both the homogeneous (“diffuse and shared”) and heterogeneous (“spatially selective”) correlation schemas are characterized by a correlation strength coefficient (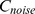, see Eq. 17 and 18) ranging from 0 (no imposed correlation) to 1 (identical synaptic bombardment for every TC cells).

We gradually increased the correlation parameter 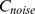 while measuring the firing correlation of TC neurons pairs in model circuits (Eq. 20). Correlations in the cortical input provoked pairwise spike correlations in the thalamic layer (Fig. 4B, left). The two correlation schemas did not affect the population in a similar way. The homogeneous correlation schema induced an homogeneous distribution of pairwise spike correlations across the population (Fig. 4B, upper right; distributions shown for 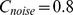 indicated by the vertical dashed line) while the heterogeneous correlation schema induced a bimodal distribution characterized by strong spike correlations only in a subset of TC cells (only receiving shared inputs) and no correlation other than the chance level for the remaining cells (only receiving independent inputs) (Fig. 4B, lower right).

Next, we compared the spiking activity of the cortical response with the average thalamic response. We measured both the firing rate and the spiking variability during fully synchronized (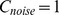) or uncorrelated (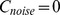) cortical bombardment in model circuits. Although the correlations introduced in the synaptic bombardment across cells did not affect the mean and standard deviation of the cortical input nor the average response of individual TC cells, it modulated both the firing rate (Fig. 4C) and the coefficient of variation of the cortical response (Fig. 4D). In the uncorrelated paradigm, the cortical firing rate remained lower than its thalamic input. In contrast, full correlation of the synaptic bombardment increased the firing rate of the cortical cell and equaled it to the firing rate of the TC cells. Similarly, the spiking variability depended upon the level of correlation of the synaptic bombardment. Note that variability in the cortical discharge was the largest during the uncorrelated paradigm.

We then explored the impact of the synaptic bombardment correlation on the efficiency of the global retinocortical information transfer for both correlation schemas (Fig. 4E). We found that synaptic bombardment correlations injected at the thalamic level strongly decreased the TE of sensory signal transfer. The TE decrease was progressive resulting in a graded decoupling of the retinal stimulation and the cortical response. The starting and ending points were identical for both correlation schemas, only the rate of variation due to correlation increase were different in the two paradigms, being more linear for the homogeneous correlation schema than for the heterogeneous one. Full correlation of the synaptic bombardment (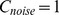) was still permissive for signal transfer albeit TE was 76% lower than that measured for uncorrelated bombardment (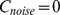).

We investigated further how the synaptic bombardment correlation across the thalamic population affected the transfer of sensory information in individual TC cells. We calculated the TE for partial information transfers between the retinal input and a TC cell response (retinothalamic TE_partial_) and between a TC cell input and the cortical response (thalamocortical TE_partial_). The synaptic bombardment correlation was varied using the homogeneous schema ensuring symmetric variations in all TC cells. A single thalamic cell was thus arbitrarily chosen to calculate partial retinothalamic and thalamocortical TEs.

The partial thalamocortical TE was improved by the correlations present in the CT synaptic bombardment (Fig. 4F, squares), an effect which at first look seems opposed to the decrease observed on Figure 4E for the global retinocortical TE. This apparent contradiction is due to the fact that two different things are measured. The global retinocortical TE measures the strength of the coupling between the retinal input and the cortical response, reflecting the capability of the whole circuit to transfer retinal sensory information to the cortical cell. The partial thalamocortical TE specifically focuses on the coupling between a TC cell input and the cortical response. Increases in correlation levels of the CT synaptic bombardment degraded the retinocortical coupling while it improved the thalamocortical coupling. In contrast, the partial retinothalamic TE was unaffected by correlation changes in the CT inputs (Fig. 4F, circles). The latter finding obeys an invariance principle in the first order statistics seen by individual TC cells (note here that correlation changes across the TC cells do not affect the mean or the standard deviation of the synaptic bombardment conductances).

**Figure 5:**
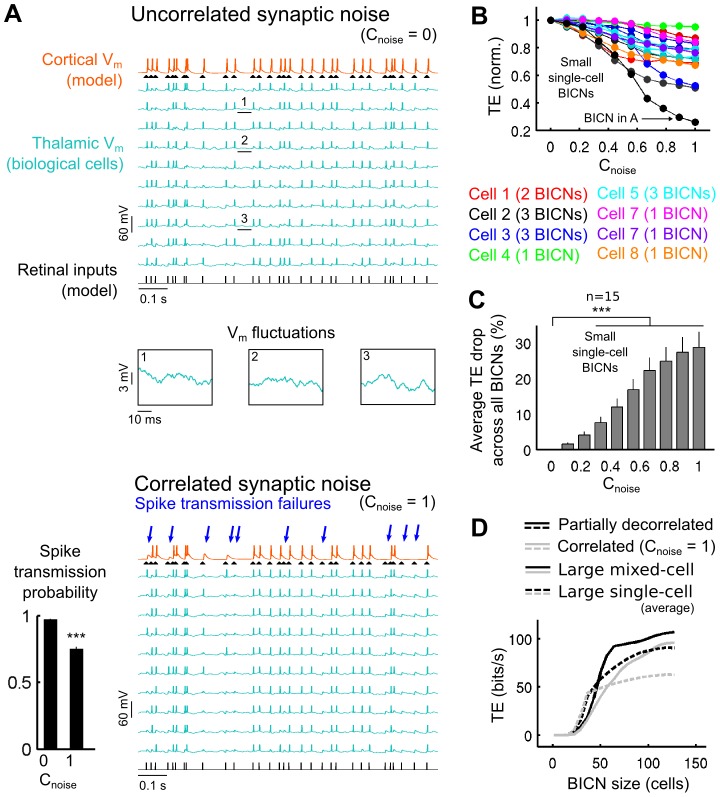
Decorrelation of the corticothalamic synaptic noise boosts retinocortical signal transfer in BICNs. A. Top. Illustration of voltage traces for a small single-cell BICN (indicated by an arrow in B) receiving uncorrelated synaptic bombardment. Insets. Zoomed sections of the biological TC cells membrane voltage fluctuations. Bottom. Same BICN as above receiving a correlated synaptic bombardment. Numerous spike failures are observed compared to the uncorrelated synaptic bombardment. The lower left bar graph shows the mean (± SEM across all spikes) retinocortical spike transmission probability for both the uncorrelated and correlated conditions. B. Transfer efficiency as a function of the synaptic noise correlation strength in small single-cell BICNs (see Methods) normalized relative to the respective uncorrelated condition of each BICN (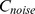 = 0). Each curve represents a different BICN with varying synaptic bombardment correlation strength. The correlation was varied using the heterogeneous schema. Curves with similar colors represent BICNs built from the same biological TC neuron. C. Average TE drop for all small single-cell BICNs (± SEM across all BICNs) as a function of the synaptic bombardment correlation strength. D. TE measurements for large mixed-cell BICNs and average TE for large single-cell BICNs of varying size for both correlated and partially decorrelated conditions (see Methods).

The simultaneous decrease of the global retinocortical TE and increase of the partial thalamocortical TE are both explained by a stochastic resonance effect between the synaptic bombardment noise and the response of every TC cells at the whole population level. The synaptic bombardment can sometime provoke spikes in TC cells which are decoupled from the retinal input if the fluctuations are depolarizing, and conversely prevent the generation of TC spikes in response to retinal events when the fluctuations are hyperpolarizing. Therefore, in presence of highly synchronized corticothalamic noise, spikes provoked or prevented by the synaptic bombardment in TC cells are amplified simultaneously in the whole thalamic population, resulting in a more uniform response. High pairwise spike correlations among TC cells reveal this uniformity (Fig. 4B). The uniformity of the thalamic responses across TC cells further lead to increased spike transmission errors (Fig. 5A, spike transmission failures in the correlated condition) which is precisely what degrades the coupling (i.e. the global TE) between the retinal input and the cortical response. Another related consequence of the cortical input synchronization is the elevation of the thalamocortical synchrony, which boosts the thalamic population drive of the cortical cell. This effect is reflected by an increased partial TE value between any sampled TC cell and the cortical cell.

In summary, dissecting the analysis of information transfer properties at different levels of the circuit reveals that decorrelation in the synaptic bombardment of the corticothalamic input induces a stochastic facilitation process between the retinal input and the target cortical cell which only emerges at the whole thalamic population level (resulting from the collective action of all TC cells). This facilitation process optimizes the efficiency of the global retinocortical transfer of information when TC cells membrane potential fluctuations are decorrelated.

### Impact of synaptic noise temporal correlations in reconstructed biological networks

Running biological exploration in parallel with simulation is a useful strategy for the refinement of the model parameters and allows checking their consistency in a biological situation. The explorations of the parameter space with the model suggested the use for BICNs of small fluctuation amplitudes for the synaptic bombardment. This strategy led to the important finding that small membrane fluctuations in individual biological cells (SD = 1–1.4 mV in model cells and 0.9–3.5 mV in biological cells after removal of spikes; see Fig. 1C,D and Fig. 5A) —that may go unnoticed in *in vivo* recordings— have a strong effect on the transfer of information when considering the whole TC cell population synaptically converging to a same cortical cell. The use of BICNs (detailed below) also led to the observation that membrane properties of biological cells is an important element for information transfer, not only a the level of the single cell, but especially at the level of the circuit. Note that these experimental findings may not be fully captured by simulations due to inherent limitations of any model.

We examined more in-depth the impact of correlations in the synaptic bombardment by conducting information transfer analysis on BICNs built from the recordings of 8 TC biological neurons (see Methods). BICNs were analogous to the model circuits tested in Figure 4. Pseudo-TC cells activities in BICNs replayed membrane potential traces recorded in biological TC neurons. The neurons were recorded in dLGN slices of rats and mice and stimulated with an input identical to the one used in model TC cells, using patch and sharp intracellular electrodes and the dynamic-clamp technique (see Methods).

First, we built 15 “small single-cell” BICNs (see Methods) each made of 10 pseudo-TC cells derived from the recordings of a single biological cell. We varied the correlation of the synaptic bombardment across the pseudo-TC cells as was previously done in the model circuits. The correlation parameter (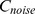) ranged from 0 to 1 using the heterogeneous thalamocortical correlation schema. Illustration of recording sequences from a BICN is shown for both the uncorrelated (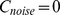) and correlated (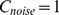) conditions (Fig. 5A). Close examination of the voltage fluctuations in the uncorrelated condition revealed notable differences, reflecting variations in the injected synaptic noise conductances (Fig. 5A, insets). Variations in the voltage fluctuations were also present in the correlated condition, albeit much smaller, reflecting solely the trial-to-trial variability intrinsic to the biological TC cell. We found that, when compared to the uncorrelated condition, the correlated synaptic bombardment failed to elicit a number of cortical spikes in response to the retinal input (Fig. 5A, lower left bar graph). The retinocortical global transfer efficiency decreased with increasing levels of correlation in the thalamic layers (Fig. 5B), confirming the results obtained *in computo* in previous sections. The average transfer efficiency drop in the 15 BICNs became highly significant for correlation strengths larger or equal to 0.33 (Fig. 5C; p = 0.00031 for 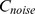 = 0.33; p = 0.000015 for 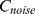 = 1; paired-sample t-test). These *in vitro* results confirmed that an increase in the synaptic bombardment correlation led to a significant decrease of the retinocortical transfer efficiency.

Next, we constructed “large mixed-cell” BICNs (see Methods) with a number of pseudo-TC cells ranging from 0 to 130. We compared the TE for correlated (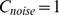) and partially decorrelated synaptic bombardments. Compared to the previous BICNs made of 10 pseudo-TC cells obtained from a single biological cell (Fig. 5A to 5C), the large mixed-cells BICNs (Fig. 5D, solid lines) were built from a collection of distinct biological cells which added cellular diversity and variability in the membrane potential fluctuations due to the differences in biological cell properties. We found that the TE was lower in the correlated condition for network sizes larger than ∼50 pseudo-TC cells, thus confirming the paradigm by which correlation in CT synaptic inputs decreases the efficiency of the sensory information transfer in the more realistic case of BICNs made of distinct biological TC cells.

BICNs offer an opportunity to explore the impact of biological cellular diversity on the transfer of sensory information. In order to suppress diversity, we built “large single-cell” BICNs (see Methods) in correlated and partially decorrelated conditions. Similarly to the small single-cell BICNs, each large single-cell BICN was obtained from the recordings of a single biological cell. Large single-cell BICNs thus differs from the large mixed-cell BICNs by their lack of cellular diversity while still maintaining the trial-to-trial variability inherent to intracellular recordings. In large single-cell BICNs, thalamic layer sizes larger than the previously described small single-cell BICN from which they are made from, were achieved by duplicating separately the activities of the pseudo-neurons. To illustrate this, a large single-cell BICN composed of 130 pseudo-cells was built upon a set of recorded sequences of activity used previously in the construction of an individual small single-cell BICN composed of 10 pseudo-cells, with each sequence being duplicated a total of 13 times. We then averaged the TE for each thalamic layer size and each correlation condition of the above large single-cell BICNs (Fig. 5D, dashed lines). We found that TE averaged for the large single-cell BICNs was lower than TE obtained for the large mixed-cell BICNs, indicating that there was a positive contribution of the biological cellular diversity in the transfer of sensory information. For a size of 130 pseudo-cells, large mixed-cell BICNs TEs were 18% and 54% higher than the average TEs for large single-cell BICNs, in the correlated and partially decorrelated conditions, respectively.

In the light of these results obtained by combined *in vitro* and *in computo* approaches, we propose that active decorrelation of background synaptic activity in the thalamic layer provides a powerful optimization mechanism —emerging from a population effect— controlling the efficiency of the retinocortical signal transfer. In this framework, each TC cell is seen as a detector of the retinal stimulation and the brain could modulate the overall transfer efficiency via the CT feedback correlation by controlling the level of independency between the individual detectors, ranging from fully synchronized (lowest information rate) to desynchronized (highest information rate). In the next section, we further investigated the impact of cellular diversity on information transfer in model circuits.

### Parametric study of cellular heterogeneity as a “decorrelation” source

In addition to the influence of synaptic inputs and the ongoing afferent activity, the putative diversity of intrinsic membrane properties encountered within a same cell class or across different cell classes due to the variety of their detailed morphology and the distribution of their ionic channels may also contribute to the decorrelation of the cells activities. We therefore investigated in model circuits to which extent intrinsic cellular heterogeneity could affect the retinocortical global information transfer.

We introduced cellular heterogeneity in our convergent networks by randomizing key intrinsic parameters reflecting the number of channels, the morphology and the integration time constant of the TC neurons (see Methods). We defined a “cellular heterogeneity” index, ranging from 0 to 1, as the amount of variation of the randomized parameters, where a value of 0 meant there was no variation at all and a value of 1 meant that randomized parameters varied up to 100% around their respective original values (Eq. 4). This randomization was repeated for each TC cell. The synaptic bombardment parameters were kept identical to those used previously in model circuits made of non-randomized model TC cells. Note that as a consequence, the bombardment was no longer optimal after randomization of the TC cells intrinsic properties and, a priori, it would require a cell-per-cell adjustment to optimally adapt the bombardment to the new properties of each TC cell. To summarize, two types of decorrelation coexisted in these new simulations, respectively of extrinsic (synaptic bombardment) and intrinsic (biophysical cellular diversity) sources.

**Figure 6:**
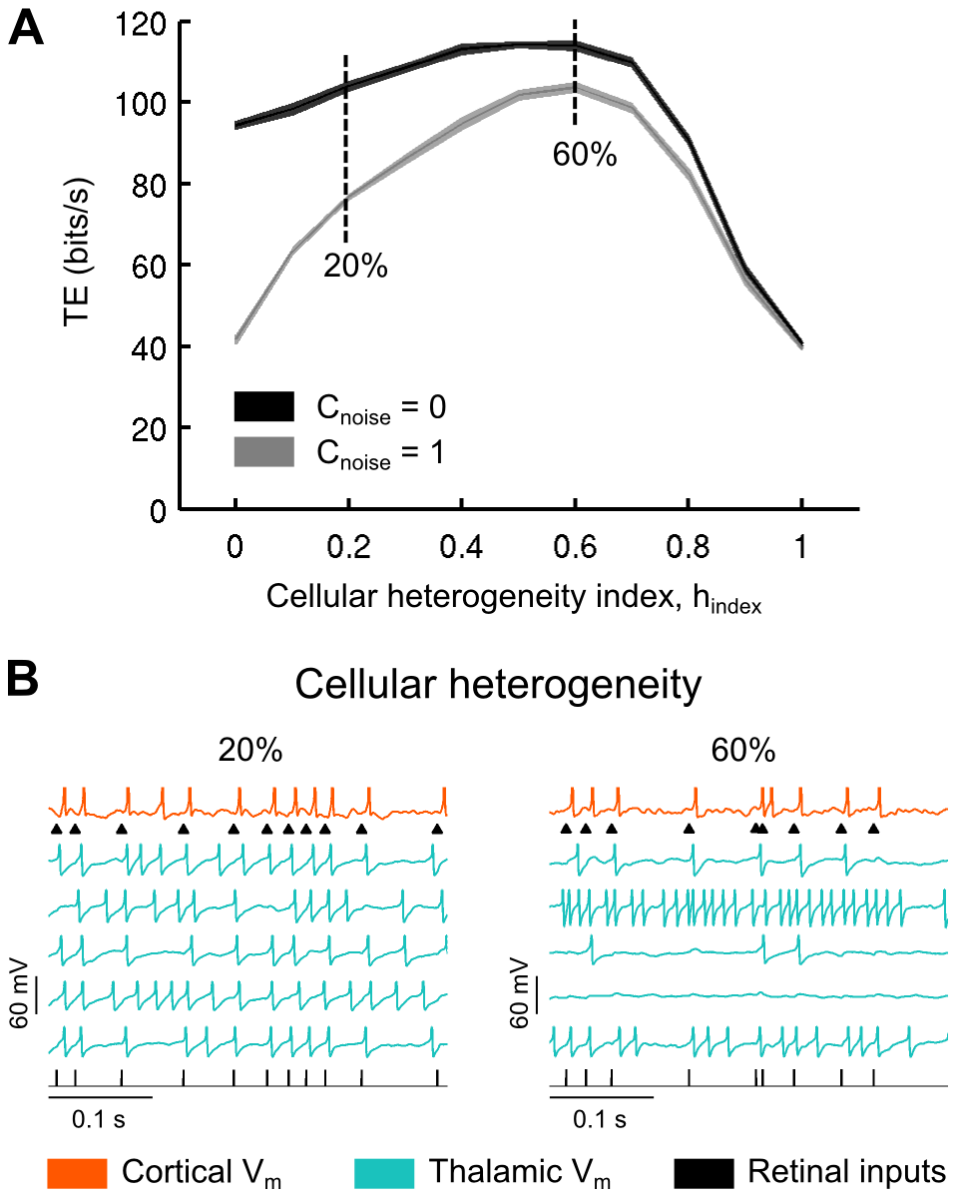
Cellular heterogeneity improves the retinocortical signal transfer *in computo*. A. Transfer efficiency as a function of the cellular heterogeneity index (see Methods) for both uncorrelated and correlated synaptic bombardment. B. Model voltage traces are shown for moderate (20%) and very high (60%) cellular heterogeneity.

Starting with pools of identical TC cells (where the cellular heterogeneity index is 0 in Fig. 6A), in both the correlated and uncorrelated CT synaptic input condition, we found that moderate to high cellular heterogeneity was associated with an improved TE, up to a maximum of 60% of variation for all parameters, after which further cell variability led to degradation of transfer efficiency. Figure 6B illustrates the activities of cells for moderate (20%) and very high (60%) cellular heterogeneities. Comparing the two curves, it is important to note that cellular heterogeneity is very effective in rescuing the low information rate resulting from the correlated synaptic activity, as was previously observed in BICNs (Fig. 5D). Cellular heterogeneity has much less effects in presence of uncorrelated synaptic activity, especially for a moderate, presumably realistic, cell heterogeneity of around 20% (see Discussion).

In summary, these simulations show the diversity of possible mechanisms through which information transfer can be controlled, and the importance of decorrelated background activity in the gating of input from the sensory periphery to cortical areas.

### Impact of coherent oscillations in the thalamic layer

We finally considered an extreme mode of correlation, present in the brain in the form of widespread synchronized oscillations of various but specific frequencies, that are known to impair signal transfer during sleep [36], [37], absence epilepsy [38], promote loss of consciousness [39] and show reduced magnitude during focal attention [40]. We investigated to which extent such oscillation-induced correlations imposed in the convergent structure of the thalamic network would affect signal transmission.

**Figure 7:**
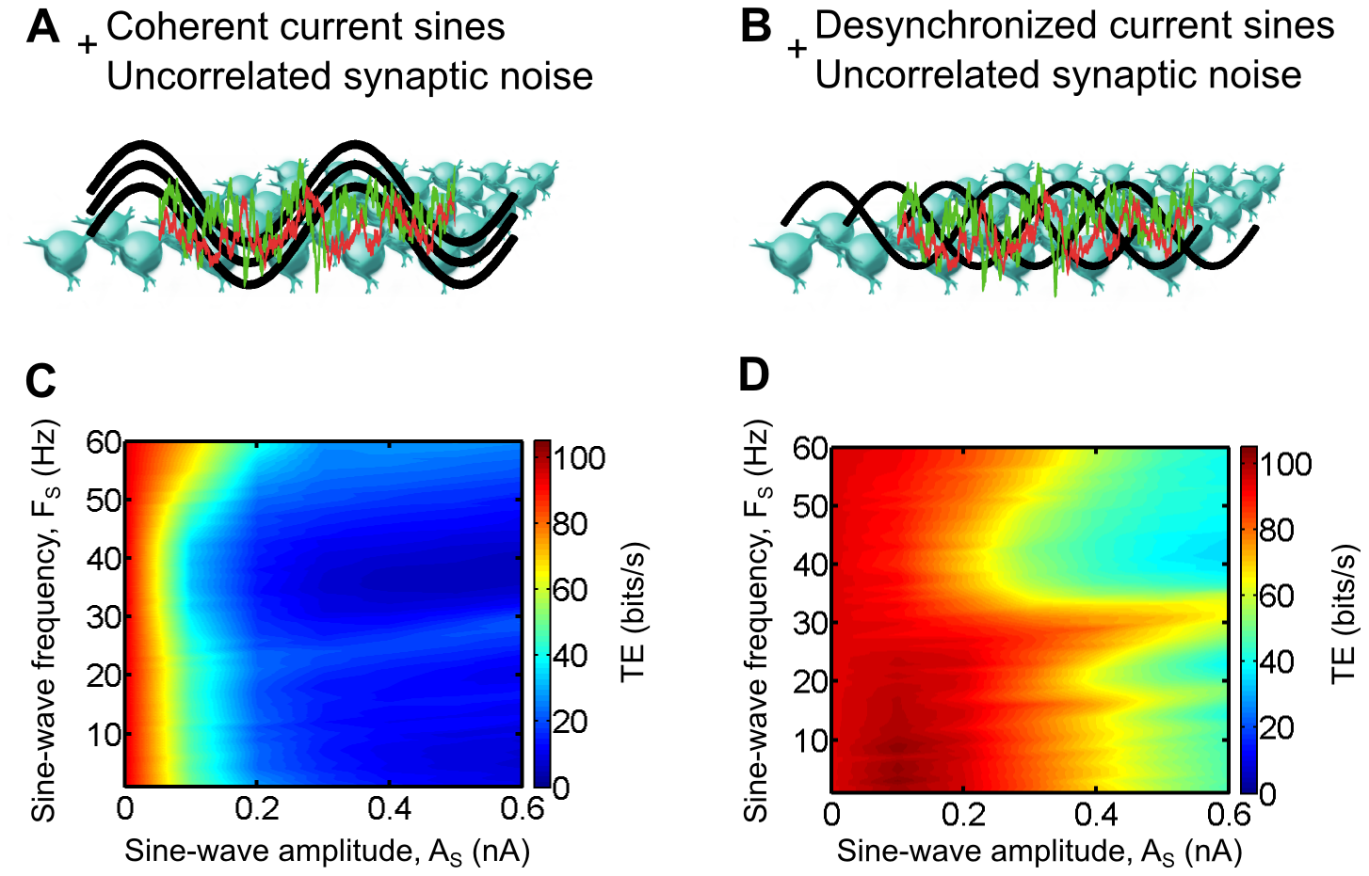
Impact of thalamocortical oscillations on the retinocortical information transfer efficiency *in computo*. A and B. Sine-wave currents of varying amplitude and frequency were injected to every model TC cells in addition to retinal inputs and uncorrelated synaptic bombardment. The current oscillations were either coherent (same phase for every TC cells) or desynchronized (phase evenly distributed in the thalamic population). C and D. Transfer efficiency for both conditions shown in A and B, respectively.

We induced oscillations in the thalamic layer of model circuits by injecting sine-wave currents of varying amplitude (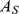) and frequency (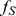) to every TC cells (Eq. 14) in addition to a cell-independent synaptic bombardment (no imposed correlation, 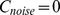). In a first case, the phases (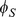) of the sine-wave currents were identical across all TC cells which resulted in coherent oscillations in the thalamic layer (Fig. 7A). In a second case, the oscillations were desynchronized by a homogeneous distribution of the sine-wave phases across the thalamic population (Fig. 7B).

We found that imposing coherent oscillations resulted in a large decrease of the TE for the full range of tested frequencies, as soon as the oscillation amplitude became large enough (Fig. 7C). In contrast, the desynchronized oscillations were not as effective to decrease the TE. For particular oscillation frequencies, larger amplitudes, by at least three fold, were required to achieve a similar drop compared to the coherent oscillations (Fig. 7D). Moreover, the coherent oscillations achieved the same TE decrease for every tested frequencies while the desynchronized oscillations were more effective in dropping the transfer efficiency for the 30–60 Hz (gamma) frequency range. Changing the retinal input discharge frequency did not affect the shapes of the graphs (data not shown).

Consistent with recent reports showing task-dependent changes of the oscillatory synchrony in the alert animals (see Discussion), we propose that one important role of the cortical feedback is to modulate the spatial coherence of the thalamocortical oscillatory activities in order to regulate the efficiency of the retinocortical sensory transfer. Combined with a dynamic modulation of the first-order statistics of the CT input (classical single cell gain control), these mechanisms could be used by the brain to actively filter the information conveyed by the retinal ganglion cells to the cortical areas, reflecting both attentional processes and active stimulus filtering under the supervision of higher areas in the brain.

## Discussion

In this paper we have quantified the impact of the corticofugal synaptic bombardment on information transfer at the scale of the whole thalamic population presynaptic to a cortical neuron, both in biological iteratively constructed networks and model circuits. Cortically-induced fluctuations of the synaptic conductances were mimicked with a stochastic process and injected in the biological cells through dynamic-clamp. The main finding reported here is that the circuit simulating the convergence of thalamic neurons onto a common target cortical cell, constitutes a distributed array of input sources which are ideal targets for a top down control. Cortically-controlled stochastic facilitation in individual thalamic cells add up to form an emerging signal filtering property at the network level that promote accurate retinal spike transfer to cortex. We show that this property is critically controlled by the number of TC cells involved simultaneously in the convergence, the statistics of the cortically-driven synaptic bombardment, and the level of correlation imposed across membrane potential fluctuations of the TC cells.

Simulations limited to a realistic range of biophysical parameters in synaptic weight and EPSP amplitude show that an optimal number of ∼90 TC cells was best adapted to favor the transfer of sensory information to the convergent circuit topology, which is characterized by weak TC synapses and a high degree of TC convergence. Cortically-induced thalamic voltage fluctuations could be adjusted to control the thalamic spike synchronization window thus sharpening the cortical spike-triggered average response and the efficiency of the sensory input transfer. Most importantly, we found that cortical input coherence was a key factor controlling the sensory signal information transfer efficiency to the target cortical cell. Simulation of coherence increase across TC cells by imposing additional correlated random fluctuations or coherent voltage oscillations in their membrane potential gradually degraded the sensory signal transfer. In contrast, a relatively high amount of retinal afferent synchronization was critical to ensure efficient transfers.

Our approach calls however for some reservation: in its detailed implementation, the present BICN circuit does not implement in full the feedback between cortex and thalamus, since the simulated cortical input to the thalamic relay cells is not updated by the ongoing output activity of the thalamocortical stream. However, our aim was to explore the functional impact of a parametrized cortical input signal whose statistical structure has the “color” of a “realistic” cortical feedback. One could still object that such added feedback could simulate as well a contextual noise at the thalamic level. Nevertheless, our working hypothesis posits that the “color” of this noise is dictated by the corticothalamic loop. There is indeed biological evidence supporting our theoretical framework: in contrast to the assumed distributed nature of the cortical feedback, it is well established that ongoing activity of intra-thalamic origin (as observed *in vivo* [41] and *in vitro* [42] in the deafferented slice) has a strong rhythmic dominance [43], [44]. In order to test the impact of such oscillatory noise source, we imposed voltage oscillations in the TC cells and found that coherent oscillations have the property of reducing the sensory signal information transfer whereas desynchronized oscillations remain permissive.

We propose that the synaptic gating of sensory information in the thalamus may rely on transient and spatially-focalized modulations of the coherence level of the contextual cortical feedback.

### Optimal size of the convergent circuit

The convergent synaptic organization of the thalamocortical circuit forms the structural kernel of our feedforward model. This rather simple topology of projecting relay neurons in the visual thalamocortical system [3] has not attracted the attention it deserves perhaps because of the yet unsolved technical challenge of identifying and record simultaneously all neurons belonging to the same convergent circuit. In this paper we reveal that this convergent topology might be essential for information transfer, first because, in a somewhat trivial way, it allows the recipient cortical cell to integrate sensory EPSPs evoked in all the relay cells simultaneously, and second, in a less obvious way, because the concomitant synaptic bombardment exerted by the descending corticothalamic feedback can result in stochastic facilitation of the feedforward input lines.

We found the number of thalamocortical cell involved in the convergent network to be a key parameter controlling information transfer. An exhaustive exploration of the parameter space revealed that the efficiency of the transfer reached a peak for a population of ∼90 relay cells, with rather weak synapses (∼1 mV EPSP), whose individual recruitment would never trigger per se a cortical spike. For larger population sizes, little or no gain was observed, indicating a saturation of the impact of the convergent afferent circuit.

These results are coherent with findings obtained *in vivo*. Thalamocortical inputs on layer 4 stellate neurons are thought to be among the strongest in the neocortex. However, recent *in vivo* data show that individual synapses are weak (EPSP ∼0,5–2 mV) and that a subnetwork of at least ∼30 inputs needs to be synchronously active to drive the firing of a single cell in the visual cortex [31], [45], [46] and in the somatosensory cortex [47]. Our model is based on data from the visual thalamus and by exploration of the parameter space we found that statistically at least one third of the 90 cells are required to fire simultaneously in order to elicit a spike in the target cortical neuron. It should be noted that despite their different functional specialization, the topology of converging TC circuits to V1 and S1 seem to retain the similarity of having numerous and synchronous convergent inputs to layer 4 cortical cells (estimated to ∼85 in S1 [47]; and range between the extreme values of 15 to 125 in V1 [2], [3]).

It is important to note that the optimal size of the convergent circuit we reported here was directly obtained from numerical explorations performed on the retino-thalamo-cortical model circuit. Put together, the above results led us to the suggestion that the value of ∼90 TC cells is of significance not only for the visual thalamic network but potentially for similar feedforward multi-layered networks as found in other sensory modalities. Nevertheless, although the model circuit parameters are extremely consistent and thoroughly constrained with biological data, it is likely that more exhaustive simulations are needed in order to estimate the actual optimal value of the population size adapted to other network topologies (with different synaptic convergence and divergence ratios), cellular properties and input statistics (which differ significantly across sensory modalities).

### Synchrony detection and spike-timing in thalamocortical convergence

The activity of local groups of cells with neighboring receptive fields can be significantly correlated if the visual input itself has strong spatial and temporal correlations (for a review see [48]), as it is the case with natural scenes [49]–[51]. LGN cells with overlapping receptive fields of the same type (ON-center or OFF-center) often fire spikes that are synchronized within 1 ms *in vivo* [1] and their precise correlation was found to be of considerable importance [52] in the coding of visual information. In our model circuit, synchronization of the LGN inputs to the cortical cell was directly controlled by the retinal spike synchronization parameter in a biologically realistic retinothalamic stage, where multiple retinal ganglion cells were connected through both convergent and divergent processes to the TC cells. In accordance with the works cited above, we found that a relative synchronization of the retinal afferents was critical to convey an efficient transfer to the cortical neuron (Fig. 2C).

Interestingly, the thalamocortical convergent circuit was adapted to detect synchrony of the LGN cells. Successful propagation of retinal spikes to the cortical cell required the LGN spikes to fall within a ∼10 ms time window. This estimate corresponds to twice the peak half-width of the spike-triggered average for the optimal regimes in Figure 3C and is consistent with the “spiking opportunity window” for thalamic spikes [53], thalamic synchronization tuning resulting from adaptation [54] and retinogeniculate paired-spike transmission enhancements [55], [56].

In contrast with the importance of the synchronization level of the retinal inputs, the precise timing of individual LGN action potentials within this spiking opportunity window was not a critical factor for an efficient information transfer to the cortical cell. We found that in the presence of background synaptic noise, information transfer remained relatively resistant to the deleterious effect of retinal and thalamic spike-time jitters. In a demanding test in which increasingly large delay jitters were randomly applied to the feedforward circuit (Fig. 2C,D), transfer efficiency decreased by less than 20% for delay jitters up to 3 ms in either the retinal or the thalamic inputs. This suggests that the deleterious effect of retinocortical propagation variability (estimated to 1 ms for the retinothalamic transmission [57] and to 0.4 ms for the thalamocortical transmission [58]) on signal transfer can be easily overcome by the gating effect of the corticothalamic feedback on the TC cell population.

Whether correlations among the retinal ganglion cells are strong enough to drive synchronously their thalamic targets remains a matter of debate. A possibility is to consider multiple arrays of small intermingled thalamocortical convergent networks, such as studied here, each capable of detecting and relay specific sets of synchronous retinal ganglion cells. In this framework, each set of synchronously active retinal ganglion cells could represent a distinct feature of the visual scene and convergent networks involved in the synchrony detection of the latter sets could propagate meaningful representations of the visual space to cortical layer 4. This intuitive proposition should be tested in larger scale computer simulations.

Feedforward inhibition in the cortex is another feature that could facilitate information transfer. Circuits with strong FFI can selectively gate synchronous over asynchronous inputs ([32]). This predicted that in our model —in which synchrony of thalamocortical inputs to cortex is paradoxically favored by uncorrelated corticothalamic noise— a strong cortical FFI could sharpen the synchrony of excitatory input and thus increase information transfer. We found that transfer efficiency in the quiet and optimal regimes was mostly unaffected for a large range of biologically realistic parameters. However, FFI could partially rescue information transfer in the saturated regime (Fig. S4).

### The challenging task of modeling the activity of layer 6 corticothalamic neurons

Considering their massive projections to the thalamus and to other cortical layers, and of their strong synchronizing role during sleep (see final part of Discussion), it has been a long-standing enigma that layer 6 (L6) neurons are largely unresponsive or fire at low rate in the lightly anesthetized [59] and in the awake animal [60]–[62]. Nevertheless, Swadlow [61] predicted that “the high degree of receptive field specificity of L6 neurons implies that action potentials of such neurons carry a high significance”. By depolarizing L6 neurons, Kwegyir-Afful et al. [63] unraveled that the vast majority (∼80%) of L6 neurons respond to whisker deflection. One can argue that even if their individual firing is low, the overall spiking activity is high because of the large number of cells involved [62]. Recent studies suggest behavioral circumstances in which corticothalamic neurons could be engaged. Feedback from the cortical area MT (V5) to layer 6 of V1 is particularly interesting because it has the potential to influence the feedback to the LGN directly (see [18]). Similarly, during voluntary whisking, sensory transmission in whisker/barrel thalamocortical circuits may be modulated according to specific activation patterns that are generated in the motor map [20]. The authors found that L6 corticothalamic neurons responded more robustly to whisker deflections when motor cortex activity was focally enhanced. Therefore, it is critical that future studies of L6 in S1 be done in the behaving animal and engages motor cortex.

The activity of L6 may influence cortical sensory responses directly through intracortical projection and indirectly through corticothalamic feedback projections. In the visual system, the activity of L6 neurons and the relative contribution of the two different L6 neurons projections, as been recently clarified by Olsen et al. [21]. First, they show in the mouse visual cortex that L6 neurons are spontaneously active and that their activity increases during visual stimulation (Fig. 1D in [21]). Second, when L6 activity is artificially increased by broad optogenetic photostimulations or by full-field visual stimulation, it has a suppressive effect on other cortical layers and on the dLGN. Suppression in the dLGN is at odds with studies enhancing the activity of corticothalamic feedback projections through focal pharmacological manipulation of L6 neurons, which typically reported a facilitation of functionally or topographically aligned thalamic neurons overlaid by broader surround suppression [13], [18]. To reconcile these findings, Olsen et al. [21] discussed that their results are consistent with pharmacological models, because full-field visual stimulation involves the spatial summation of individual inhibitory surrounds and will result in a net suppressive effect of the corticothalamic feedback projection.

### Single cell versus population coding

Looking at the effect of the cortically-induced synaptic noise on TC cells responsiveness, we found important distinctions when considering either the isolated cell or the mesoscopic organization level of an assembly of thalamic cells.

In individual TC cells, input-output transfer efficiency can be measured by evaluating the spiking response probability of the neuron to individual excitatory retinal-like synaptic inputs. Such a probabilistic input-output curve defines the neuronal responsiveness over a wide range of inputs and is characterized by its slope, or gain. This transfer property has been shown to be efficiently modulated by the dynamical interactions between the inputs and the synaptic bombardment-induced membrane voltage fluctuations [19], [64], a phenomenon linked to stochastic resonance [65], [66]: increases in membrane potential variance resulted in an enhancement of the probability of spike generation to small amplitude conductance inputs, which were previously ineffective in the absence of noisy background bombardment.

In this previous approach, synaptic noise controlled the cell responsiveness in a probabilistic manner, and the repetition of trials of similar inputs was necessary to average the response over time and build up the full description of the input-output transfer response. In the real brain, the need for immediate response makes trial averaging impossible and there must be mechanisms responsible for the rapid extraction of the probability function underlying neuronal responsiveness. We show here that such a process can be embedded at a higher level of integration, where a target cortical cell can decode probabilistic signal integration distributed in the thalamic convergent circuit. A corollary of this property emerging at the population level is that it might be difficult to unravel in experiments in the awake animal. A possibility would be to identify and record extracellularly a great number, if not all, of the thalamic cells involved in the convergent circuit and their target cortical cell. A very challenging alternative would be to record simultaneously many TC cells intracellularly in order to unravel the correlation level of the synaptic bombardment.

### The amplitude and fluctuations of background synaptic noise determine information transfer

Several non-exclusive mechanisms may contribute to a permissive action on sensory transfer in the thalamus. The first one is the well-described neuromodulation of membrane properties of relay cells. The activity of brainstem afferents releases neurotransmitters in the thalamus (acetylcholine, noradrenaline, etc.), and results in depolarization of thalamic relay neurons out of the voltage range in which rhythmic oscillations are prevalent and promote a state of single spike activity [67]. We had previously shown in hybrid circuits that application of noradrenaline increased both retinal spike transfer efficiency and reliability to cortex [68]. This neuromodulatory effect is however acting slowly. In contrast, the cortical control of thalamic transfer efficiency by a tunable mixed excitatory and inhibitory synaptic background activity, as proposed in the present study, presents several advantages over the modulation by slow neuromodulators: It is dynamic, fast and topographically precise.

Systematic exploration of the parametric space defined by excitatory and inhibitory conductances (at the level of the thalamic population) led us to define an “optimal noise”. This noise level is characterized by rather small fluctuation amplitudes (compared with the previous works cited above), corresponding to irregular and weak fluctuations around a balanced excitation/inhibition regime. The amplitude of the voltage fluctuations in biological (SD = 0.9–3.5 mV) and model neurons (SD = 1–1.4 mV for low and high conductance states, respectively) fell in the lower end of the distribution of fluctuation amplitudes for which responsiveness was enhanced in isolated relay cells recorded *in vitro* (see Fig. 1C in [19]).

We defined low and high conductance states that differ by their total CT synaptic conductances but shared high information transfer capabilities, provided that the ratio of the excitatory to the inhibitory components of the synaptic bombardment was optimally adjusted. The distinction between low and high conductance states is nevertheless important. 7 to 16% of synapses on relay cells are from retinal afferents [69], [70] and ∼60% from the CT feedback. The remaining ∼30% of relay cell inputs originates from other thalamic areas, from indirect CT input whose cortical cells are not located in the striate cortex and not contacted by LGN cells [9] and from the diffuse neuromodulatory afferents mentioned above [67]. These additional inputs may occlude the modulatory effect of the dedicated CT feedback on the TC cells via a change in the conductance of the cell membrane. In this situation, an efficient modulation of the TC cells may not be successfully achieved by a low conductance state feedback and would rather require a high conductance CT feedback input.

In terms of conductance proportion, optimization of the transfer efficiency by means of mutual information analysis led to total corticothalamic input averages of 20.85 nS (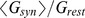 = 2.5) and 33.36 nS (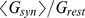 = 4) in the low and high conductance states, respectively. Adding the retinothalamic AMPA synaptic weight, 12.5 nS (estimated from [71], [72]), and assuming the resulting quantities account for 70% of the total input of the TC cells as seen above (the remaining ∼30% inputs of thalamic origin are not modeled in the present study), the retinal input can be estimated to 26% and 19% of the total input conductance of the TC cells for the low and high conductance states, respectively. It is important to note that, as shown by these ballpark estimates, the proportion of the retinal input to the total input of the TC cell was not a fixed constant in our model, but rather depended on the conductance state of the cortical input. Given the dynamic nature of the cortical feedback, it is probable that this ratio varies *in vivo*. Other factors such as the contribution of other inputs originating from thalamic areas or the statistical structure of the stimulus could also play a role on this ratio.

Interestingly, depolarizing effects of neuromodulation during waking may modulate the effects of corticothalamic inputs. We have tested this effect by depolarizing the thalamic cells with a positive constant current, and this shifted the optimal response ridge seen in Figure 3A towards lower values of excitatory conductances, and increased the sensory signal transfer efficiency in absence of synaptic bombardment (Fig. S3B).

These observations point out that the functional role of the CT feedback could be ensured in a variety of physiological or pathological synaptic contexts, by simply adjusting the contextual bombardment mean amplitude and fluctuations, including the ratio of excitation and inhibition, over a wide-range of conductance states. An intriguing question is whether such a property could be involved in adaptation of the thalamic circuitry to disruptive effects resulting from peripheral or central neural dysfunctions, such as age-related macular degeneration, stroke or phantom limb pain. While phantom sensation and tinnitus might result from a local deafferentation of thalamic circuit from sensory inputs in the somatosensory system and auditory system respectively [73] (comparable to the situation in Fig. 4F where the thalamocortical partial transfer is highest but decoupled from the sensory afferents), age-related maculopathy (ARM) is characterized by a progressive loss of central vision resulting from retinal impairments of localized area in the fovea [74]. Thalamic cells in register to the macula are consequently decoupled from the normal retinal information flow. However, in early stage of the disease, when the macula degeneration is still limited, the visual symptoms are inconspicuous [75] and one may ask if compensatory mechanisms such as an adaptation of the corticofugal activity, are already at work in the corresponding thalamocortical circuitry. Clinical recovery training protocols for ARM patients aim indeed at restoring a “displaced fovea” in the areas adjacent to the macula, where the retino-thalamo-cortical circuitry is intact. We speculate that in addition to plasticity mechanisms, the clinical paradigm may benefit from the training itself in shifting attention. We propose that the synaptic resonance process reported in the present study may be operating to the benefit of the trained patient, when he has learned successfully to redirect the focus of corticofugal synaptic bombardment on the thalamic representation of a displaced fovea, at the healthy periphery of the degenerated macula.

When considering the CT feedback as the result of a cortical computation in response to various stimuli, it has been proposed that part of the cortical function is devoted to predict future sensory inputs and constantly readjusts its output to optimally reflect the afferents-driven cortical representations [10]–[12], [18]. Hence, we could expect the dense and continuous activity of the CT feedback to strongly modulate the thalamus by causing retina-unrelated synaptic events and spikes in the TC cells in time register when retinal spikes are expected by cortical higher areas.

This resonance behavior could account for the modulation of sensory transfer in the thalamus during attention. Attention typically amplifies neuronal responses evoked by task-relevant stimuli while attenuating responses to task-irrelevant distracters [76], [77]. Clear attentional effects have been demonstrated in the thalamus of the monkey performing an attentive task [15], [78]. When the animal focused his attention on a visual clue located in the receptive field of thalamic relay neurons, these cells had their firing increased by 12–21%. In these experiments, individual relay neurons were recorded extracellularly, giving no information on the intracellular mechanism responsible for the attention-dependent increased firing. We can only speculate in accordance with the authors that this increase is likely resulting from a change in the balance and strength of L6 cortically-driven synaptic inputs. Note also that our working hypothesis differs from that formulated by [79] where the attentional state modulates the classical input/output gain of neurons, i.e. the slope of their psychometric curve, without affecting the membrane potential contextual fluctuations of the relay cells.

### Is (de)correlation in the converging thalamic layer a candidate mechanism for selective attention?

Most studies on the function of the corticothalamic feedback seem to assume a predictive coding role, essentially based on the precise topography, increased discharge and timing of corticothalamic projections [10], [11], [18], [80]. The mechanisms implementing selective attention at the circuit level in the thalamus might however take other forms less conspicuous than a mere increased cellular discharge, as suggested by studies in the monkey area V4 in the neocortex. Individual V4 neurons responded to attended stimuli that were not salient enough to elicit a response when unattended. This lowering of detectability threshold and increase in sensitivity was reflected in a leftward shift in the contrast-response function without a substantial increase in the firing response to high-contrast stimuli [81]. Multiple units recording in other studies revealed that spatially selective attention acts to reduce task-irrelevant correlated noise [82], [83]. The source of noise originates from slow to intermediate timescale fluctuations in firing rate that are correlated across relatively large populations of neurons and it has been suggested that the attention-dependent reductions in correlated firing could produce a far greater improvement in signal-to-noise ratio than increases in firing rate associated with attention would do [83].

How can the assumption of a precise implementation for predictive coding be reconciled with the randomness of the decorrelated synaptic noise responsible for stochastic facilitation? As already discussed above, it all depends upon the scale of organization that one considers. The concept that visual cognitive features emerge at a scale of encoding more mesoscopic than that of the neuron is a common assumption and was theorized for instance by Alan Newell (for a review see [84]).

Seemingly random at the individual cell level, synaptic noise provides precision when it is actively decorrelated at the circuit scale within the thalamocortical convergent circuit of ∼30–100 neurons. This can first be seen by comparing the traces in Figure 5A, in which the uncorrelated noise increases accuracy of spike relay, as well as the information content (Fig. 5B). At the cellular level, we have also tested for changes in the level of randomness of the stochastic synaptic noise. This was done by injecting in each TC cell gradually increasing correlations between 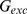 and 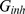 with varying correlation time lag (not to be confounded with the correlation of noise across cells). We did not observe any significant change in the signal transfer information content (see Figure S5) indicating that noise decorrelation is most effective at the scale of the thalamocortical convergent circuit. Similarly to the “rescuing” effect of feedforward inhibition in the cortex, we speculate that signal transfer of TC cells discharging in a tonic mode could be improved by correlations between 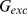 and 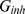.

We further investigated the effects of noise decorrelation by implementing various regimes of correlation in the synaptic bombardment across the TC cells in BICNs. Similarly to results obtained in model circuits, we found that a desynchronized (uncorrelated) top-down input was highly efficient to promote retinal signal transfer to the cortical neuron, while correlated input had the opposite effect of strongly reducing the relay. The explanation is that when the synaptic bombardment was highly correlated across the thalamic population, the TC cells membrane voltage fluctuations were nearly identical (on the basis of similar intrinsic membrane properties), resulting in an uniform response in the whole thalamic population by either amplifying or attenuating every TC cell simultaneously. For instance, when a strong depolarization due to the top-down synaptic bombardment would elicit spikes in the entire population of TC cells, the convergent structure of the circuit would then amplify the signal, and transmit a retina-unrelated spike to the cortical neuron. Therefore, correlated background inputs made the TC cells act as similar detectors and implied an all-or-nothing behavior in the convergent circuits detrimental to signal transmission. Conversely, weakly correlated or uncorrelated inputs resulted in a stochastic facilitation effect [85], possibly linked to a collective stochastic resonance mechanism [65], [66] acting independently in each TC cell. Each cell thus becomes an independent detector discerning features in the retinal signal that may not be seen by neighboring cells.

At low correlation level in the CT synaptic bombardment, a slight increase of correlation led to a large decrease in the efficiency of the retinocortical transfer (Fig. 4E) despite an almost undetectable increase in the pairwise correlations between the relay cells (Fig. 4B). These results show that differences in thalamic pairwise spike correlations, so small that they could barely be detected using dual recordings, may nonetheless strongly impact thalamocortical processing. This is consistent with a recent study in the primary visual cortex of awake macaques, showing that neurons with similar orientation tuning virtually share no correlation [86], and another study stressing the high impact of the low correlations in neural populations [87].

Background noise (de)correlation is not an all-or-none “permissive” mechanism. As seen throughout our analysis, not only can top-down synaptic inputs actively impose a state of decorrelation in the thalamic activity, characterized by high level of information transfer, they can also adjust the level of information transfer by imposing graded degrees of correlations in the circuit. Even in presence of fully correlated corticothalamic synaptic bombardment the retinocortical transfer is not entirely switched off (in other words the cortical response is only partially decoupled from the retinal input). This can be seen in the model in Figure 4E. Transfer efficiency decreases gradually from a value near 95 bits/s for uncorrelated noise, reaching a floor value of approximately 23 bits/s for maximally correlated noise. A similar tendency is obtained using a different measure as seen in the graph of spike transmission probability in Figure 5A for biological cells. Spike probability is increased from 75% for the correlated noise to near 100% for the uncorrelated noise (note that values of spike probability cannot be directly compared to those of transfer efficiency).

To conclude this section, we would like to suggest a few cognitive processes emerging in the early visual system for which a mechanism of actively decorrelated top-down synaptic bombardment could be at work.

It was proposed by Sillito et al. [18] that feedback from MT (V5) has the capacity to influence V1 and LGN cells at retinotopic locations ahead of the current stimulus location. MT receptive fields are much (up to ten times) larger than those of V1 cells. According to the authors, “a moving stimulus entering an MT receptive field, and causing it to respond, will start to drive a feedback influence that affects V1 cells at retinotopic locations ahead of and around the actual stimulus location”. Although the authors hypothesized a feedback-induced increase in cellular discharge, they did not clearly identify how top-down modulation affects cellular responses in V1 and the LGN. We propose that the permissive feedback cascading down from area MT could be implemented at precise locations in V1 and thalamus by the decorrelation of a synaptic bombardment. According to recent studies, focused attention decorrelates V4 activity in the attending monkey [82], [83]. We speculate that MT activity could be similarly decorrelated when a moving bar enters the receptive fields of MT cells, enabling top-down modulation from MT area to rapidly and precisely decorrelate V1 cells around and ahead the retinotopic positions matching the bar in movement. V1 neurons feedback input to LGN would therefore be decorrelated too, thus improving signal transfer at selective positions matching the retinotopic area covered by the activated MT cells.

Attentional modulation originating in higher-level visual areas and directing its focused action on low-level visual areas is central to the “Reverse Hierarchy Theory” [88]. It posits that the “pop-out” phenomenon (when a visual stimulus stands out from the background) is assigned to initial perception at high-level areas using their large receptive fields. This phenomenon is extremely rapid and robust, and in the case of complex objects may not incorporate fine details of the stimulus (for instance perceiving words before letters or the “forest before the trees”). Filling-in the details demands focused attention and it is proposed that later feedback reentry to low levels slowly adds details available in the small receptive fields found in primary areas. The nature of the feedback is unknown and we speculate that the decorrelation of synaptic bombardment targeted to fine features of the visual scene in the early visual system (V1 and thalamus) could play a role. A detailed schema along with in-depth explanations of this hypothesis can be found in Figure S6 and Text S1.

To summarize this key section, corticothalamic input-induced correlations in the thalamus embody yet another population emergence effect in which the overall retinocortical transfer efficiency is not modulated by a variation in the activity of single TC cells but rather by the differential integration of many TC cell responses. We propose that a stochastic facilitation process [85] emerging at the whole thalamic population level enhances the global sensory information transfer in presence of decorrelated corticogeniculate feedback bombardment. We propose further that such a process may be the basis for attentional modulation of sensory signals at the thalamic level.

### Other possible sources of decorrelation

Neurons in the brain present a wide variety of intrinsic properties and morphological characteristics. There are different thalamocortical cell-types, which may vary from cell to cell in its detailed characteristics. We therefore studied the impact of such heterogeneity, as a source of decorrelation on signal transfer in large-scale model and biological (BICNs) convergent networks.

It was recently shown *in computo* that an heterogeneous population of neurons generated more output entropy than a population made of identical neurons [89], [90]. The author's proposal that neuronal heterogeneity may improve the coding capacity of neural ensembles can be explained by the fact that heterogeneity contributes to decorrelate the population activity as discussed above, leading to an increase in the diversity of the neurons' responses at the population level (hence contributing to the output entropy increase). Note that output entropy quantifies the amount of diversity in the response while the mutual information measurements we performed characterize both the output diversity and the dependency of the responses on the input stimuli.

Our results in large-scale BICNs (Fig. 5D) and in large-scale circuit simulation (Fig. 6) extend the previous findings [89], [90]. In both cases heterogeneity of cellular properties boosted information transfer. In the model, we investigated various levels of heterogeneity by designing thalamic layers composed of heterogeneous model TC cells that were obtained after randomization of the conductances parameters of the original model TC cell. We found that information transfer kept increasing until cellular heterogeneity reached biologically unrealistic levels (some TC cells display a highly saturated discharge activity, others are silent; Fig. 6B, traces for 60% variation). This result is surprising since a number of TC cells taken individually fail to respond properly to the retinal inputs. It should be noted that this “rescuing” effect of heterogeneity was particularly prominent in presence of correlated synaptic noise, corresponding to low information transfer. In contrast, the cellular heterogeneity rescuing effect was largely occluded by the voltage variability imposed by the synaptic bombardment in the uncorrelated situation. This aspect is interesting for several reasons. First, input synaptic variability is a dynamical process governed by the presynaptic activity of thousands of neurons and is capable of adapting rapidly, perhaps instantaneously, to the needs of sensory transfer. In contrast, alteration of cellular biophysical properties is a slow adaptive process [91], which is ill-suited to achieve fast and reversible dynamical regulation of sensory transfer. Second, despite the cell-to-cell differences, synaptic bombardment statistics (mean and standard deviation of the excitatory and inhibitory synaptic noise conductances) did not need to be tuned in a cell-specific manner in order to provide an efficient transfer. Instead, tuning the statistics as a whole for the “average cell” in the heterogeneous population resulted in a higher transfer efficiency compared to the same tuning in an homogeneous population. This apparent discrepancy between results obtained in the isolated cell and in the circuit is an indication that distributed changes imposed in the statistics of the synaptic noise in individual cells may have a fundamental and unpredictable impact at the level of the whole thalamic population.

Put together, these results bring new insights about the possibility of a combined interplay between the synaptic bombardment and the heterogeneity in the intrinsic determinants of cellular excitability. Both may contribute to the decorrelation of input signals in order to reduce the redundancy of the signals and maximize information transfer, in a passive manner via intrinsic properties variability and in an active manner via background synaptic activity self-generated by the cortex.

### The coherence of thalamic oscillations controls information transfer

The top-down correlations we implemented among the TC relay cells concerned the whole frequency spectrum of the synaptic noise conductances. Another way by which the brain imposes correlated firing in the LGN arises from oscillatory activities which drive correlated spiking during periods of depolarization synchronized among the TC cells. Oscillations are rather stereotyped in frequency and amplitude, lack the broadband variability of the statistical structure of the cortical noise and are widely present in the thalamocortical system during wakefulness and sleep. There is a growing body of evidence that they could be associated to sensory flow filtering and attentional modulation.

From wakefulness to sleep, a variety of rhythms have been reported in the thalamus. In relaxed wakefulness, the electroencephalogram (EEG) exhibits robust rhythms in the α band (8–13 Hz), which decelerate to θ (2–7 Hz) frequencies during early sleep [44], [92], followed by the 10–14 Hz spindles waves and the slow (<1 Hz) rhythms during non-REM sleep ([93]; for a recent review see [94]). Spindles are perhaps among the best-understood synchronized oscillations generated endogenously in the thalamocortical system during slow wave sleep ([42]; for a review see [43]). Spindles are known to be spatially correlated in the thalamocortical system, and lose their coherence after decortication in the cat, demonstrating the involvement of the CT feedback in the correlating process [41].

We proposed earlier that spindle oscillations, studied at the single-cell level, had the property of imposing a temporal decorrelation of retinal cell input and thalamic relay output, resulting in the functional disconnection of the cortex from the sensory drive [68]. This idea was confirmed recently in human in a study showing that the amount of spindles correlated with sleep robustness: people having more spindles were more likely to stay asleep in noisy situations [36]. Consistent with this report, it was later proposed that synchronized oscillations in the alpha band are part of an active attentional suppression mechanism aimed at ignoring irrelevant or distracting information [95]. Signal decoupling by means of synchronized oscillations is most likely to reach its maximum impact in situations of anesthesia or epilepsy. A theoretical study based on human EEG recordings suggested that the thalamocortical coherence during the alpha rhythm produced by Propofol, a short-acting hypnotic agent, is a generative mechanism for the loss of conscious sensory experience [39].

Conversely, during wakefulness, the waning of correlations and/or coherent oscillations, and in particular the decrease of power in the alpha band [40], seem to be associated with attention. An analogous phenomena was reported in the primary somatosensory cortex of humans for the mu rhythm [96].

This view is supported by the present work showing that the decoupling effect of synchronized oscillations culminates when implemented at the population level, in the ∼90 cells of the convergent thalamocortical circuit. We found *in computo* that coherent thalamic oscillations in a broad range of tested frequencies effectively reduce the retinocortical signal transfer efficiency compared to desynchronized oscillations.

However, an opposite effect was reported in a study where coherent thalamocortical oscillations in the beta range (15–30 Hz) and increases in both the LGN and the primary visual cortex gamma power were observed in cat local field potential (LFP) recordings during focused attention [97]. The authors proposed that enhanced beta activity within the primary visual cortex and LGN might be an electrophysiological correlate of the attentional mechanism that increases the gain of afferent visual information flow to the cortex.

This discrepancy may only be apparent. While a growing body of evidence [36], [39], [40], [82], [83], [96], including the present results, points towards the importance of decorrelation for an improved sensory flow, we may consider the idea that a putative active filtering mechanism for attentional modulation should not only favor the relay of relevant information but simultaneously and actively reduce the non-relevant information for the current task and context. In this model, active decorrelation, that was shown theoretically to emerge from recurrent network dynamics [98] and feedforward inhibitory circuitry during sensory stimulation [99] would favor at any point of space and time a given sensory information stream while simultaneously shutting down other non-relevant streams by imposing correlations and/or coherent oscillations.

These different hypothetical schemas lead us to consider the thalamus as an addressable array of massively intertwined input lines converging onto cortex, among which only a limited number would become elected at a given time depending on the resonance of the local sensory input with the cortical prediction: the top-down feedback could act not only as a “searchlight” but could authorize the efficient transfer of the sensory drive throughout an ever changing landscape of “hot spots” immersed in a network otherwise decoupled from the external drive.

## Methods

### Ethics statement

All *in vitro* research procedures concerning the experimental animals and their care adhered to the American Physiological Society's Guiding Principles in the Care and Use of Animals, to European Council Directive 86/609/EEC and to European Treaties Series 123 and were also approved by the regional ethics committee “Ile-de-France Sud” (Certificate 05-003). Animals used in these experiments have been bred in the Central CNRS Animal Care at Gif-sur-Yvette (French Agriculture Ministry Authorization: B91-272-105) under the required veterinary and National Ethical Committee supervision.

### *In vitro* preparations

*In vitro* experiments were performed on 300–350 µm-thick slices from the dLGN of rat thalamus in either interface style or submerged recording chambers. Wistar rats, 4–6 weeks old for sharp recording and 14–25 days old (CNRS, Gif-sur-Yvette) for patch recording were anesthetized with sodium pentobarbital (30 mg/kg) or inhaled isoflurane before decapitation, craniectomy and brain removal. Slices were prepared with a vibratome in a solution in which the NaCl was replaced with sucrose while maintaining an osmolarity of 314 mOsm and were maintained in the interface recording chamber at 34–35°C. During recording, the slices were incubated in slice solution containing (in mM) 126 NaCl, 2.5 KCl, 1.2 MgSO_4_ (sharp recording) or 2 MgCl_2_ (patch recording), 1.25 NaHPO_4_, 2 CaCl_2_, 26 NaHCO_3_, and either 25 or 10 dextrose (for interface and submerged chambers respectively) and aerated with 95% O_2_-5% CO_2_ to a final pH of 7.4. 30 minutes to 2 hours of recovery were allowed before intracellular recordings. Sharp micropipettes were filled with 1.2–2 M potassium acetate and 4 mM KCl and had resistances of 80–110 Mohm after bevelling. Patch electrodes (tip resistance: 2–3 Mohm) were filled with a solution containing (in mM): 135 K-gluconate, 0.1 CaCl_2_, 5 MgCl_2_, 1 EGTA, 10 HEPES, and 4 Na-ATP, 15 phosphocreatine and 50 units/ml creatine phosphokinase (pH adjusted to 7.3 with KOH, osmolarity 290 mOsm). The liquid junction potential (+10 mV) was systematically corrected at the beginning of the recording. Patch recordings were performed at 32°C. Access and series resistances were constantly monitored and data from neurons with more that 20% of changes from initial value were discarded.

The dynamic-clamp technique [26]–[28] was used to inject computer-generated conductances in real neurons. When using sharp electrodes, dynamic-clamp was coupled with an Active Electrode Compensation (AEC) method that we developed and validated recently *in vivo* and *in vitro* [100]. AEC allows the removal of electrode noise from intracellular voltage recordings in real time. The dynamic-clamp software is based on a custom ADC/DAC program used for data acquisition and analysis (Elphy2, developed at UNIC by Gérard Sadoc) and is interfaced with a Real Time-NEURON environment [28], in which the NEURON simulator v6.0 [101] was modified and recompiled to run under the INtime stack (TenAsys), a kernel driver enabling real time operation under Microsoft Windows OS. Stimulation protocols were run in real time with the acquisition card at 10 kHz.

Dynamic-clamp was used to insert retinothalamic inputs and cortically-induced synaptic noise in thalamic neurons. The synaptic noise was simulated using excitatory and inhibitory fluctuating conductances generated as independent stochastic processes unless stated otherwise (see following sections) and mimicking the effect of thousands of stochastically glutamate- and GABA-releasing synapses [102], as detailed below.

We selected 8 thalamic neurons from 5 animals for which intracellular recordings were very stable during long periods of time in order to perform a sufficient number of sequential conductance injections. Each of these sequences had to be long enough to get a large number of spikes for the purpose of calculating the mutual information. These neurons had a resting potential ± standard error of measurement (SEM) of −66 mV and an input resistance of 88 MΩ when recorded with sharp electrode in an interface chamber (1 neuron) and of −71±2 mV and an input resistance of 338±48 MΩ when using patch pipettes in a submerged chamber (7 neurons). All cells showed classical rebound burst discharges accompanied by low-threshold calcium spikes (LTS) upon repolarization after hyperpolarization.

### Circuit modeling

The circuits were modeled under the NEURON simulator and are described in the Results. Cortical and thalamic model neurons are based on single-compartment Hodgkin Huxley type models developed in previous modeling studies [103], [104]. The cortical neuron is based on a pyramidal layer 4 cell and the thalamic cells are based on a thalamocortical relay cell. The model retinal cells consisted in random spike-train generators mimicking the discharge pattern of an ON-center Y cell. Model parameters for cell passive properties, synaptic currents and synaptic bombardment are summarized in Table 1 Cellular models, current kinetics and BICN implementations are described in the sections below. The model files are available on ModelDB website, accession number 150240.

### Retino-thalamo-cortical circuit topology

The model circuits were composed of 1 or 15 retinal cells (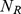), 1 to 240 TC relay cells (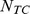) and one recipient cortical cell. Synapses were either excitatory-type or inhibitory-type and mimicked AMPA (α-amino-3-hydroxy-5-methyl-4-isoxazolepropionic acid) and GABA_A_ (gamma-aminobutyric acid type A) mediated current flows in the postsynaptic compartments, respectively.

Unless otherwise mentioned, a common retinal spike train was simultaneously fed to the whole thalamic population. In this case, each TC cell was coupled to the retinal cell through a single excitatory synapse.

In some cases, multiple retinal lines were simulated (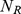 = 15). Each retinal cell projected to 4 TC neurons and each TC neuron was contacted by 2 retinal cells [1]. For each TC cell, the sum of the synaptic weights coupled to the incoming retinothalamic synapses was identical to the single synaptic weight used in the single retinal cell version of the circuit. The original retinothalamic synaptic weight (12.5 nS, [71], [72]) was splitted between the two retinothalamic synapses in the proportion of 75% and 25%, giving synaptic weights of ∼9.4 nS for one synapse and ∼3.1 nS for the other synapse.

The degree of convergence between thalamic and cortical neurons is well quantified [2], [3]. The many-to-one thalamocortical convergence was implemented by connecting each TC cell to the cortical cell through a single excitatory synapse.

Conduction delays were neglected since retinothalamic synapses have been shown to be synchronized within a millisecond [1], [57] and TC propagation delays exhibit a very low variability [58]. No plasticity rules were implemented in our models.

### Cellular model and intrinsic currents

Model neurons were described with the following equation
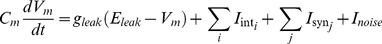
(1)where 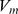 is membrane potential, 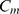 is the capacitance of the cell, 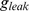 is the leakage conductance, 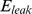 the leakage reversal potential, 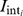 is an intrinsic current, 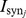 is a synaptic current, 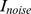 is a fluctuating synaptic current and 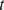 is the time variable. By convention, positive currents were directed towards the soma and provoked depolarization of the membrane potential.

Intrinsic currents (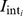) were described by the generic form
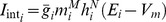
(2)where the current is expressed as the product of respectively the maximal conductance, 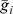, activation (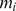) and inactivation variables (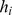), and the difference between the membrane potential 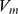, and the reverse potential 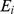. Activation and inactivation gates follow the simple two-state kinetic scheme introduced by Hodgkin and Huxley (1952)
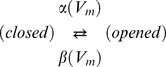
(3)where 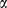 and 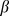 are voltage-dependent rate constants.

The set of intrinsic current was different for cortical and TC model neurons. For each current, parameter values were obtained from matching the kinetic model to voltage-clamp data. The intrinsic currents used here and references for more details were: I_T_ and I_h_ in TC cells [105]–[107], I_M_ in cortical cell [108] and for both cell types, I_Na_−I_K_ currents responsible for action potentials [109]. In TC cells, Ca^2+^ dynamics and all other parameters were identical to a previous model [105].

Resting input conductance (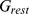) and resting membrane potential (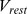) were set to 33.4 nS and −70.6 mV respectively for the model cortical cell and 8.34 nS and −74.3 mV for the model TC cell.

Synaptic currents (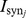) are described in the “Synaptic currents” section. Fluctuating synaptic current input (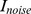) is described in the “Synaptic bombardment model” section.

### Cellular model randomization

To build up a cellular heterogeneity in the model circuits, we randomized some of the built-in parameters characterizing the TC model cells. The randomized parameters were generated from
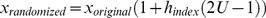
(4)where 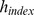 is the “cellular heterogeneity” index, 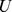 is a random number drawn from a uniform distribution on the unit interval and 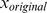 is the original value of the parameter. The randomized parameters thus varied in random proportions. The cellular heterogeneity index controlled the maximum amount of variation. Each TC cell was driven by its own set of parameters.

Randomized parameters included calcium, sodium and potassium maximal conductances of the I_T_, I_h_ and I_na_−I_K_ currents (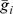), the membrane leak conductance (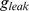) and the membrane capacitance (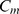).

### Synaptic currents

Synaptic interactions are mediated by conductance-based synaptic currents described by
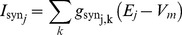
(5)where 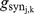 is the synaptic conductance for the spike 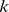 and 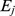 the synaptic reversal potential. Spikes elicited in the post-somatic compartment unitary conductance patterns of the form
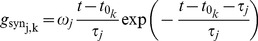
(6)where 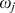 is the synaptic weight of the synapse reflecting the peak conductance amplitude, 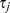 is the time to the peak amplitude and 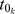 the time of the spike 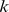. Prior to a given spike (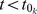), 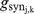 is set to 0. For clarity, excitatory and inhibitory synaptic parameters were termed by the suffixes “AMPA” and “GABA”, respectively. This conductance pattern is illustrated in the inset of Figure 1.

Retinothalamic and thalamocortical synapses were excitatory-type. Feedforward inhibition in the cortical cell involved inhibitory-type synapses (see next section).

Excitatory synaptic parameters were set to 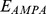 = 0 mV and 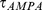 = 1 ms in both cortical and thalamic model neurons. The synaptic weight 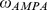 was set to 12.5 nS for retinal EPSPs in TC cells [71], [72] and 2.33 (biological estimate), 7 or 21 nS for thalamic EPSPs in the cortical cell for the 90 (biologically realistic size), 30 and 10 TC cells version of the circuit, respectively [31].

### Feedforward inhibition to the cortical cell

Feedforward inhibition was implemented by coupling an inhibitory synapse to the thalamocortical excitatory synapse. In addition to the excitatory synaptic current, each TC spikes triggered an inhibitory synaptic current in the cortical cell. Inhibitory synaptic parameters were set to 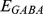 = −75 mV and 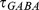 = 2 ms. The synaptic weight 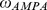 was varied from 0 to 10 nS. A positive time lag was introduced to delay the IPSPs, 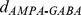, and varied from 0 to 10 ms.

### Synaptic bombardment model

In addition to the massively feedforward pathway cascading from retina to cortex, we added a corticothalamic synaptic bombardment to the TC cells, operating in a highly distributed way. In some cases, we also added a cortical bombardment to the cortical cell. The synaptic bombardment was composed of two fluctuating conductances, excitatory 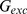, and inhibitory 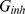, and is determined by
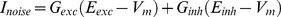
(7)where 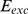 = 0 mV and 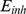 = −75 mV are the reversal potentials for excitatory and inhibitory conductances, respectively. Each CT input synaptic conductance (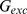 and 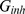) was described by a stochastic equation of the type
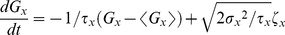
(8)where 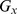 stands for either 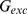 or 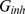, 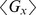 is the mean conductance, 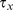 is the correlation time, 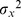 is the variance of the conductance and 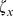 is a Gaussian noise of zero mean and unit variance. These equations are identical to the dual Ornstein-Uhlenbeck process hypothetized in [110] and reflects through a Gaussian distribution the total conductances seen by a neuron permanently bombarded by thousand of synaptic events.

Synaptic noise mean conductance values were normalized relative to the rest conductance of each neuron. Normalization relative to the rest conductance ensures that the synaptic conductances will produce a similar voltage deflection regardless of the intrinsic properties of a cell *in vitro* or *in computo*. We thus defined the “conductance amplitude” as 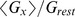 for both the excitatory and the inhibitory components of the synaptic bombardment. In order to keep the amplitude of the synaptic noise conductances fluctuation deflections proportional to the mean level of the conductances, we defined the “conductance variation ratio” as 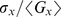 similarly to the conductance amplitude. Note that, as a consequence of this normalization, a conductance amplitude value of 0 also nullifies the conductance fluctuations.

For numerical explorations, the conductance amplitude of the synaptic bombardment was varied from 0 to 3 for both excitatory and inhibitory components, translating to 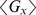 ranging from 0 to 25.02 nS in Figure 3A. The conductance variation ratio was varied from 0 to 1 corresponding to 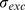 ranging from 0 to 12.51 nS and 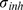 ranging from 0 to 8.34 nS in Figure 3B (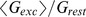 = 1.5 and 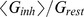 = 1.0).

The total cortical input conductance was expressed as
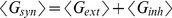
(9)and varied between 0 and 50.04 nS. Similarly, we normalized the total conductance 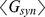 to the rest conductance of the TC cells. The normalized total cortical input conductance (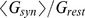) ranged from 0 to 6 in our numerical explorations.

The parameters 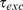 = 2.7 ms and 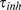 = 10.5 ms were adjusted to match the power spectrum of synaptic conductances resulting from thousands of randomly releasing synapses [110]. The mean (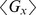) and variance (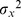) of the conductances injected in TC neurons were adjusted such as to optimize the transfer efficiency of the convergent circuit (Fig. 3, see Results). For the cortical neuron, 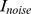 was set to 0 (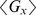 = 0 and 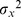 = 0).

### Retinal stimulation

The retinal stimulation mimicked the discharge pattern of an ON-center Y cell. This pattern is characterized by a 30 Hz gamma 3 distribution [29], [30] and is described by
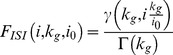
(10)where 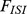 is the cumulative distribution function of the spike-time interval *i*, 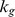 the shape parameter, 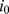 the mean interspike interval parameter, *γ* the lower incomplete gamma function and Γ the gamma function. The mean interval (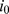) was set to 0.33 ms (30 Hz), the shape parameter (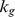) was set to 3 and the scale parameter was set to 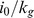.

When multiple retinal lines were simulated, the level of synchronization of the retinal afferents was parametrically controlled by modulating the number of retinal inputs replaying a common pattern. This was implemented by designing 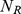 independent retinal activity input patterns, where 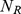 is the total number of simulated retinal lines (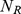 = 15). To increase the synchronization of the retinal inputs, the number of independent retinal inputs was reduced so that some of the retinal inputs remained independent while the others replayed the same retinal input pattern, 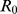. The synchronization of the retinal afferents was expressed as
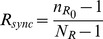
(11)where 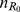 is the total number of retinal lines replaying the same input pattern 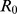 and ranged from 1 to 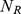. A retinal synchronization (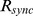) value of 0 meant there was no forced synchronization among the retinal lines and a value of 1 meant the retinal afferents were all synchronized.

Spike-time jitters were also used to desynchronize the timing of the retinal spikes in the TC cells. This implementation is described in the next section.

### Spike-time jitters

In some cases, an ad-hoc spike-time jitter was introduced so that each retinal or thalamic spike was affected by a different time jitter independently of the other spikes (Fig. 2C and 2D, see Results). Large retinal spike-time jitters led to desynchronized retinal inputs among TC relay cells and large thalamic spike-time jitters led to desynchronized thalamic inputs in the cortical cell. The spike-time jitters were randomly drawn for each spike from an exponential distribution described by
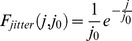
(12)where 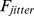 is the cumulative distribution function of the spike-time jitter *j* and 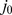 is the mean value parameter. The mean spike-time jitter (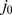) ranged from 0 to 10 ms. A value of 0 means the jitters are null (control value). Positive jitters are obtained for 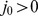.

### Constant current and current sines

We injected constant and sine-wave currents to thalamic cells. The constant current is simply described by a constant
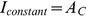
(13)ranging from 0 to 0.6 nA.

The sine-wave current is characterized by
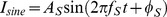
(14)where 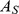 is the amplitude of the current ranging from 0 to 0.6 nA, 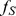 is the frequency of sine ranging from 0 to 60 Hz and 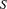 is the phase. The phase of the oscillation was either 0 for every TC cells, which was referred as the “coherent oscillations” condition, or uniformly distributed from 0 to 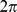 in the thalamic population, referred as the “decorrelated oscillations” condition. No current offset was applied resulting in an average 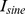 current of 0 nA. These currents were added to the membrane potential equation of the model neurons (Eq. 1).

### Temporal correlation of the synaptic bombardment

Correlation between the excitatory (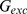) and the inhibitory (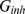) components of the synaptic bombardment were controlled by the 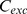 correlation parameter and the 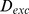 correlation time lag parameter. Correlated Gaussian noises 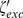 and 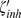 were expressed by
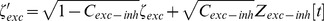
(15)and
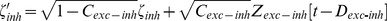
(16)


where 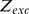 is a vector of independent Gaussian noises of zero mean and unit variance which are also independent from 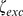 and 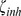. The Gaussian noises 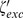 and 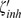 had identical statistics to 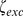 and 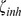, respectively, and were injected in Equation 8 as a replacement to the original Gaussian noises 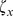.

Correlation of the synaptic bombardment across TC cells is described in the next section.

### Thalamocortical correlation schemas

TC membrane potential correlations among the whole thalamic population were induced by the CT synaptic bombardment and controlled by the 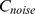 correlation parameter, ranging from 0 for cell-independent cortical inputs to 1 for common cortical inputs in the whole thalamic population. Two correlation schemas were considered.

The first schema correlated homogeneously the entire thalamic population such as
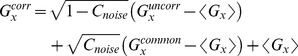
(17)where 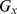 stands for either 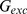 or 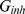, 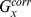 is the resulting correlated conductance noise, 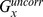 is an uncorrelated conductance noise specific to each TC cell, 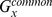 is a common conductance noise identical in every TC cells and 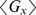 is the mean conductance. This expression assumes that 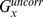 and 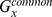 have identical mean and variance so that the resulting 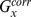 has identical statistics. This schema was denoted as the “homogeneous” correlation schema.

In the second schema, a subset of the population received common cortical inputs (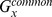) while the rest of the population received cell-independent cortical inputs (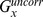). Hence, the first subset of the thalamic population had fully correlated cortical inputs. Other TC cells had no imposed correlation. We denoted this schema as the “heterogeneous” correlation schema and its correlation strength 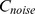 was defined by
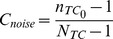
(18)where 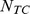 is the total population size of the thalamic layer and 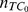 is the number of TC cells receiving the common cortical inputs patterns 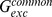 and 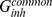 and ranged from 1 to 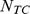. Note that the definition of 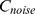 in the heterogeneous schema is similar to the definition of the retinal synchronization parameter, 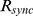 (Eq. 11).

### Biological iteratively constructed networks

BICNs [25] offered a way to explore the voltage dynamics of the thalamocortical convergence *in vitro*. We simulated the heterogeneous correlation schema of the synaptic bombardment in three types of BICNs. In all of BICN types, the model TC cells were replaced by activity patterns of biologically recorded relay TC cells. Building a BICN involved a two-steps procedure. First, biological TC neurons were sequentially recorded under various conditions described below. Second, the recorded membrane potential traces were integrated off-line in the model circuit. In the resulting hybrid circuit, each pseudo-TC cell replayed a corresponding recorded voltage trace to simulate the synaptic convergence of the hybrid thalamic layer onto the model cortical cell. Biological TC cells input was composed of artificial retinal inputs and synaptic bombardment injected through dynamic-clamp.

Biological TC cells were recorded 10 times with the same realization of a synaptic bombardment and 10 times with independent realizations of a synaptic bombardment, accounting for a total of 20 recorded voltage traces. The same retinal stimulation was fed during each of the 20 recorded sequences, and the mean and standard deviation of the synaptic noise conductances were kept identical across the recordings. The individual fluctuation patterns of the synaptic noise differed from one recording to another for the 10 independent synaptic bombardment sequences and the same set of 11 distinct synaptic bombardment patterns (1 common pattern and 10 independent patterns) were used in every biological cells. Each set of 20 recorded sequences in the same biological cell was termed a “sequence set”, and was thus composed of 10 correlated pseudo-cell activities (common synaptic noise) and 10 decorrelated pseudo-cell activities (independent synaptic noise). In some cases, we could repeat this procedure 2 or 3 times in a single biological TC cell. From these recordings we built a “small single-cell” BICN type, a “large mixed-cell” BICN type and a “large single-cell” BICN type.

The small single-cell BICN type was based on a thalamic population of 10 pseudo-neurons with thalamocortical AMPA synaptic weight optimized for this size (the optimal value was found for model circuits and is shown in Fig. 2A and 2B by the light-gray curves). We built one BICN of this type per sequence set obtained as described above, accounting for a total of 15 BICNs (cell 1 to 8 in Fig. 5B). For each small single-cell BICN, we constructed off-line 10 hybrid thalamic layers mixing sequences recorded under the same synaptic noise and sequences recorded under independent realizations of a synaptic noise in the proportions 1–9, 2–8, …, 9–1 and 10-0, respectively. These hybrid thalamic layers were thus characterized by a different correlation strength coefficient as defined for the heterogeneous correlation schema and ranged from 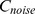 = 0 to 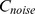 = 1 (incremental step is 1.11). Note that the first thalamic layer mixing sequences in the proportion 1–9 has an effective correlation coefficient of 0 because the 9 independent realizations of a synaptic noise were also independent from the common synaptic noise that was used multiple times per cell.

The large mixed-cell BICN type had a thalamic population size ranging from 0 to 130 pseudo-neurons. The thalamocortical AMPA synaptic weight was always set to its biological value, optimized for a biologically realistic population of 90 TC cells. To build a large mixed-cell BICN of size *N* in the correlated condition (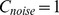), we combined in a hybrid thalamic layer *N* sequences chosen randomly among all of the sequence sets recorded in patch-clamp (cell 2 to 8 in Fig. 5B) under the same realization of a synaptic bombardment, accounting for a total 13 sequence sets and 130 correlated pseudo-cell activities. In this correlated condition, all pseudo-TC cells shared the same realization of a synaptic bombardment. Similarly, we built large mixed-cell BICNs of size *N* in the “partially decorrelated” condition (see below) by randomly combining *N* sequences recorded under one of the ten independent realizations of a synaptic bombardment (i.e. the remaining 130 decorrelated pseudo-cell activities). In the latter BICNs, there were only 10 distinct synaptic noise sequences repeated a maximum number of 13 times, hence resulting in a partial decorrelation of the synaptic noise across the TC cells. It was not possible to calculate the 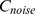 parameter in the partially decorrelated condition because the resulting correlation schema differed from both the homogeneous and the heterogeneous schemas previously described in this study.

Finally, the large single-cell BICN type was a mixture of the two previous types of BICN. Large single-cell BICNs were similar to the large mixed-cell BICNs in respect to their population size range, their thalamocortical AMPA synaptic weight and the pool of sequences included in their construction (cell 2 to 8 in Fig. 5B; patch-clamp sequences). Similarly to the single-cell BICN type, we built one large single-cell BICN per sequence set recorded in the same biological TC cell. First, for each BICN of this type, the associated sequence set was duplicated 13 times resulting in a new “duplicated sequence set” composed of 130 correlated pseudo-cell activities (common synaptic noise) and 130 decorrelated pseudo-cell activities (independent synaptic noise; note there were only 10 distinct synaptic noise sequences repeated a maximum number of 13 times as for the large mixed-cell BICN type). Large single-cell BICNs of size *N* in either correlated or partially decorrelated condition were then built as described above for the large mixed-cell BICN type by randomly combining *N* either correlated or decorrelated sequences from the associated duplicated sequence set. Finally, we averaged for each hybrid thalamic layer of size *N* and each correlation condition the transfer efficiency measured in every large single-cell BICNs as to reflect the average information transfer in the large single-cell BICNs.

### Information transfer efficiency analysis

We calculated the efficiency of the global retinocortical sensory signal transfer and partial retinothalamic and thalamocortical signal transfers by means of mutual information theoretical analysis
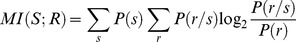
(19)where *S* denotes the stimulation, *R* the response, 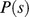 the probability of presentation of the stimulus window *s*, 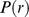 the probability of presentation of the response window *r* and 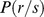 the probability to obtain the response window *r* in response to the stimulus window *s*. For retinocortical signal transfer analysis, *S* is the spiking activity of a retinal cell and *R* is the spike train response of the target cortical cell. Partial retinothalamic and thalamocortical signal transfer analysis involved the spiking activity of a chosen thalamic TC cell as the response signal *R* or the stimulation signal *S*, respectively. Stimulations and responses spike trains consisted in sequences of “0” and “1” with fixed time bin size where “0” denotes the absence of a spike in a given time bin and, conversely, “1” denotes the presence of a spike. Rarely, when more than one spike happened in a single time bin, a “1” was counted. Recorded and simulated membrane potential traces where converted to spike trains using a spike threshold of −30 mV.

To consider as extensively as possible the spatio-temporal richness of the spike trains, we looked for correlations up to 30 ms using the smallest time bin allowed by the finite size of the data. We therefore segmented *S* and *R* in windows of 30 ms. *In vitro* recording time was at least 40 seconds and *in computo* simulation time was 100 seconds. We used a 3 ms time bin for BICN transfer analysis and a 1 ms time bin for model circuit transfer analysis as a trade-off between the biological spike timing precision and the finite size of our data.

We emphasize that even if the maximum number of distinct window patterns could theoretically reach up 2^10^ for BICNs and 2^30^ for model circuits, not all configurations can occur due to the limitations imposed by the intrinsic properties of the neurons such as the after-hyperpolarization following a spike. We checked that the number of unique window patterns was small enough compared to the recording and simulation times in order to avoid the well-known “undersampling catastrophe”. We did this with the help of extensive computer simulations on biological and synthetic data by gradually increasing the time bin and decreasing the window size until the finite data set corrections as described in [111] became negligible (less than 1% of the final values). Varying the time bin and window sizes did not change the structure of the mutual information as a function of the explored circuit parameters. In the worst case it only slightly affected the overall scale of the curves. Note that correlations on a timescale higher than 30 ms cannot be excluded. However, our numerical simulations did show that around 90% of the stimulation-induced correlations in the response are included in a 30 ms window.

We then checked for residual information bias by a bootstrap procedure. We randomly paired stimulation and response window patterns and computed the mutual information from these random pairings. The information obtained in this case should be zero and is an indication of residual error so we removed this bootstrap estimate from our mutual information calculations.

Although the synaptic transmission is instantaneous in our circuit model, a time lag between *S* and *R* was set to account for the propagation time due to the integration constant of the cells. When the model relay cells were used, the lags were best estimated to 6, 4 and 2 ms for the retinocortical, retinothalamic and thalamocortical transfers, respectively. *In vitro* time lag was re-estimated for each BICN.

We also tested other methods to estimate the transfer efficiency including classical spike transfer probability and linear cross-correlation analysis (Fig. S1). In any case the mutual information analysis gave the most coherent results according to the paradigms explored in this study (see legend of Fig. S1 for more details)..

### Spike train correlation analysis

For each pair of TC cells, we calculated the Pearson's linear correlation coefficient between thalamic spike trains. The conversion of thalamic voltage responses to spike trains was done as described in the “Information Transfer Efficiency analysis” section using a bin size of 1 ms. Pairwise spike correlation coefficients are described by
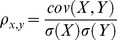
(20)where X and Y are the two spike trains, 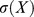 and 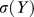 are the standard deviations of X and Y, respectively, and 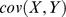 is the covariance of X and Y. Pairwise spike correlation coefficients average, 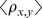, was calculated for each unique pair of TC neurons..

### Spike-triggered average analysis

For each cortical spike, a 30 ms region preceding the spike was considered. The region was cut in bins of 1 ms each. Each bin consisted in the average number of thalamic spikes 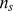 ms before the cortical spike, where 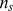 is the index of the bin on the x-axis of the STA shown in Figure 3.

## Supporting Information

Figure S1 **Methods for evaluating the sensory transfer efficiency.** A. Same as Figure 3A; for ease of comparison. B. The mutual information calculations were limited to the only knowledge of the spike count in a 30 ms window. This was done to reduce the number of symbols used in mutual information calculations and avoid any undersampling issues. The resulting transfer efficiency was underestimated but remained similar in shape to A. C. Synaptic information efficiency developed by London et al. (2002) [24] resulted in entropy values almost identical to A. D. Evaluation of the transfer efficacy, defined as the probability that a retinal spike will evoke a cortical spike in a 30 ms window following the spike. This method is similar to the classical spike transfer probability used in Wolfart et al. (2005) [19]. This analysis would alone misleadingly suggest that high efficiency is reached when the cortical firing probability is high (see the saturated regime in Figure 3C). E. Evaluation of the transfer contribution, from 0 to 1, defined as the ratio of the number of transmitted retinal spikes to the total number of cortical spikes. Low contribution values indicate that the cortical spikes are unlikely to be linked to the retinal spikes while high contribution values indicate that the cortical spikes are more likely to be evoked by the retinal spikes. White areas indicate there were not enough cortical spikes to calculate the transfer contribution. The middle area bounded by the saturation zones (efficacy and contribution≈1) in D and E is similar to the optimal red band in A. F. Classical cross-correlation analysis between the retinal and cortical spike trains with a bin size of 1 ms. The correlations were calculated using MATLAB (MathWorks) xcorr function and normalized so that the autocorrelations at zero lag are identically 1. White areas indicate that the function could not calculate the correlations and returned “NaN” values.
(TIF)

Figure S2 **Transfer functions of cortical and TC model neurons.** A. Probability that the cortical model neuron evokes a spike in a 30 ms window following an AMPA conductance event of varying amplitude. B. Same as A for a model TC cell. The probability was measured either with optimal synaptic bombardment (see low conductance state regime in Figure 3) or without contextual synaptic bombardment.
(TIF)

Figure S3 **Depolarization of the TC model neurons improves the sensory signal transfer in absence of synaptic bombardment.** A. Model circuit membrane voltage traces obtained in absence of synaptic bombardment (denoted by the arrow “0” in Figure 3A). B. Numerical explorations of the cortical input conductance amplitudes for two depolarizing constant currents. Model circuit, conductance variation ratio and analysis are identical to the ones presented in Figure 3A.
(TIF)

Figure S4 **Feedforward inhibition to the cortical cell helps sensory signal transfer in the saturated regime.** A. Transfer efficiency as a function of the feedforward inhibition GABA_A_ synaptic weight and time lag (see Methods) for both optimal regimes shown in Figure 3A. B. Similar to A for the saturated regime.
(TIF)

Figure S5 **Synaptic bombardment excitation and inhibition interplay in TC model cells.** Numerical explorations of the temporal correlations between the excitatory and the inhibitory components of the corticothalamic input at the single cell level. Transfer efficiency is plotted as a function of the excitatory–inhibitory conductance correlation strength and the inhibitory conductance time lag (see Methods).
(TIF)

Figure S6 **Speculative role of synaptic bombardment decorrelation and thalamic oscillation coherence in focused attention.** A. Visual stimulation composed of bars of various orientation. Focusing attention on a single bar (for instance vertical) will slowly segregate all other bars of same orientation from the context made of other bars of dissimilar orientation. Vertical bars are colored in brown for illustration purposes only. B. Presumed functional steps involved when focusing attention on a vertical bar. Vertical bars shown on each neuron illustrate the orientation preference. Columnar organization of V1 circuits is not illustrated although each neuron shown in this schema belong to a different orientation column. An initial decorrelation of activity in cortical area V1 is generated at the retinotopic location of the focused bar. This decorrelated activity is propagated to other regions whose orientation preference match the orientation of the focused bar. A decorrelated corticothalamic feedback is then sent to dLGN target neurons which are specifically tuned to detect features matching a bar of similar orientation. Other thalamic regions that receive no decorrelated feedback would develop synchronized oscillations. More detailed explanations of this hypothesis are provided in Text S1. C. Proposed selective attention mechanisms for sensory signal filtering. Foci of decorrelated corticothalamic activity amplify the visual streams whose features match the bars of vertical orientation while synchronized oscillations in the thalamus reduce the sensory transfer of visual features related to the bars of other orientation.
(TIF)

Text S1 **Focused attention: A synaptic bombardment decorrelation hypothesis.** Supporting evidences for the implication of synaptic bombardment decorrelation in focused attention. We propose a phenomenological model based on the segregation effect described in Figure S6. Based upon the findings of the present study, we make the prediction of the existence of dynamic functional maps of correlation and decorrelation in V1 and the thalamus. These non-classical maps provide a putative mechanism for the implementation of selective sensory attention in the thalamocortical system.
(DOC)
