# Supplementary figures and images for: Cortically-Controlled Population Stochastic Facilitation as a Plausible Substrate for Guiding Sensory Transfer across the Thalamic Gateway
Source: PLoS Comput Biol. 2013 Dec 26;9(12):e1003401. doi: 10.1371/journal.pcbi.1003401 (PMC3873227; doi:10.1371/journal.pcbi.1003401)

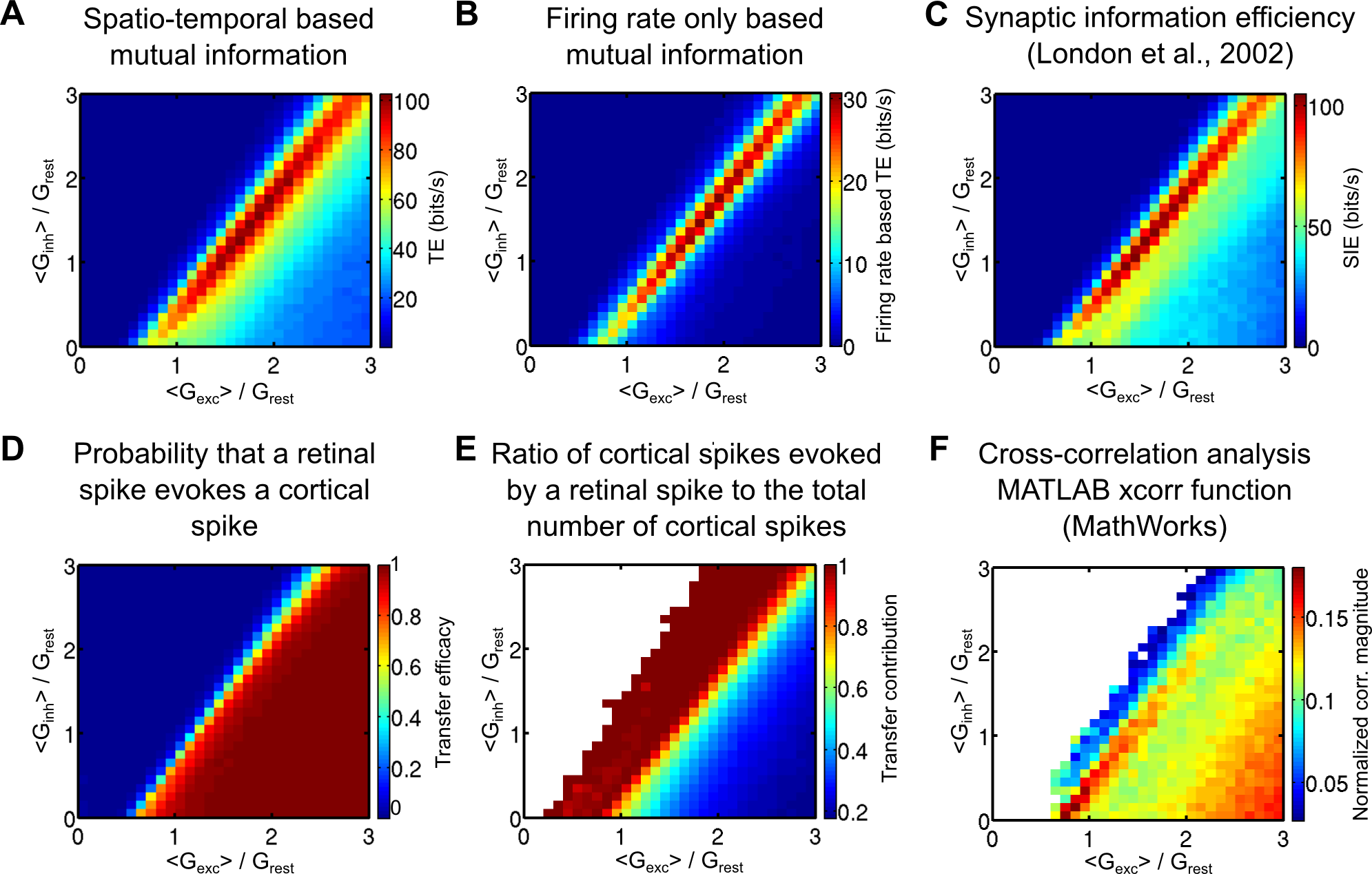

Supplement: Figure S1 — Methods for evaluating the sensory transfer efficiency. A. Same as Figure 3A; for ease of comparison. B. The mutual information calculations were limited to the only knowledge of the spike count in a 30 ms window. This was done to reduce the number of symbols used in mutual information calculations and avoid any undersampling issues. The resulting transfer efficiency was underestimated but remained similar in shape to A. C. Synaptic information efficiency developed by London et al. (2002) [24] resulted in entropy values almost identical to A. D. Evaluation of the transfer efficacy, defined as the probability that a retinal spike will evoke a cortical spike in a 30 ms window following the spike. This method is similar to the classical spike transfer probability used in Wolfart et al. (2005) [19]. This analysis would alone misleadingly suggest that high efficiency is reached when the cortical firing probability is high (see the saturated regime in Figure 3C). E. Evaluation of the transfer contribution, from 0 to 1, defined as the ratio of the number of transmitted retinal spikes to the total number of cortical spikes. Low contribution values indicate that the cortical spikes are unlikely to be linked to the retinal spikes while high contribution values indicate that the cortical spikes are more likely to be evoked by the retinal spikes. White areas indicate there were not enough cortical spikes to calculate the transfer contribution. The middle area bounded by the saturation zones (efficacy and contribution≈1) in D and E is similar to the optimal red band in A. F. Classical cross-correlation analysis between the retinal and cortical spike trains with a bin size of 1 ms. The correlations were calculated using MATLAB (MathWorks) xcorr function and normalized so that the autocorrelations at zero lag are identically 1. White areas indicate that the function could not calculate the correlations and returned “NaN” values. (TIF) [file pcbi.1003401.s001.tif]

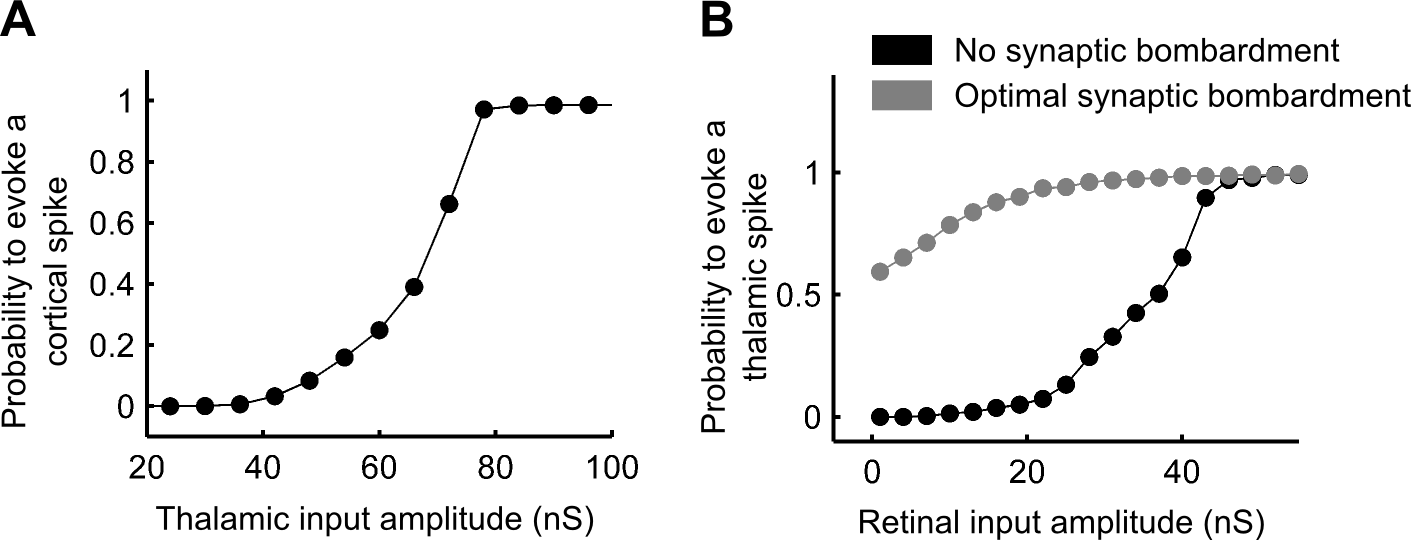

Supplement: Figure S2 — Transfer functions of cortical and TC model neurons. A. Probability that the cortical model neuron evokes a spike in a 30 ms window following an AMPA conductance event of varying amplitude. B. Same as A for a model TC cell. The probability was measured either with optimal synaptic bombardment (see low conductance state regime in Figure 3) or without contextual synaptic bombardment. (TIF) [file pcbi.1003401.s002.tif]

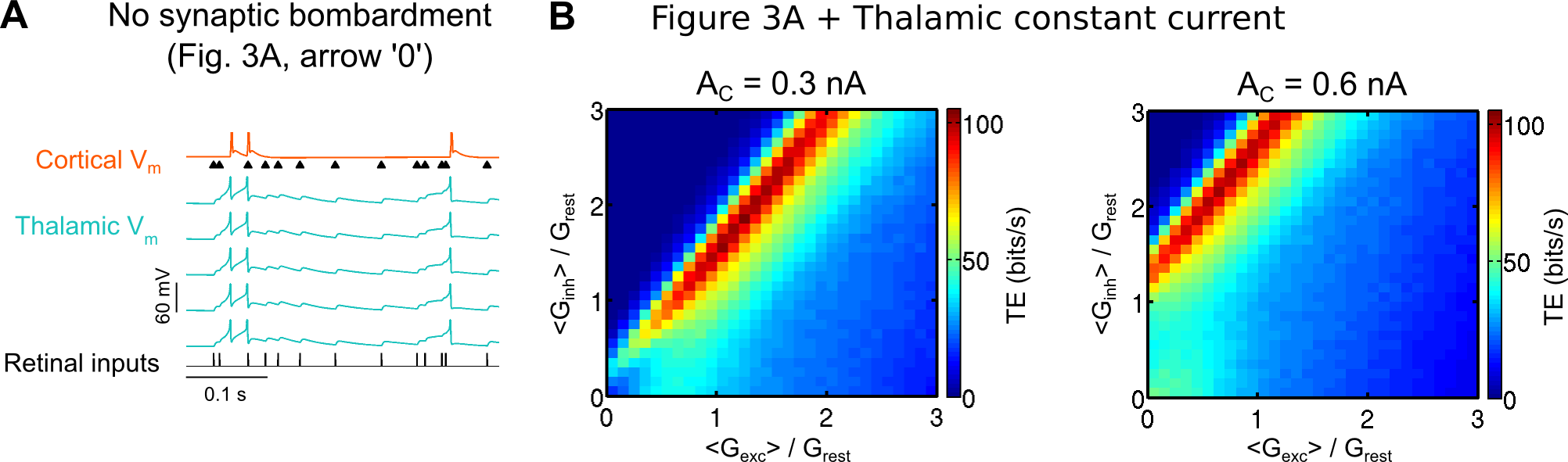

Supplement: Figure S3 — Depolarization of the TC model neurons improves the sensory signal transfer in absence of synaptic bombardment. A. Model circuit membrane voltage traces obtained in absence of synaptic bombardment (denoted by the arrow “0” in Figure 3A). B. Numerical explorations of the cortical input conductance amplitudes for two depolarizing constant currents. Model circuit, conductance variation ratio and analysis are identical to the ones presented in Figure 3A. (TIF) [file pcbi.1003401.s003.tif]

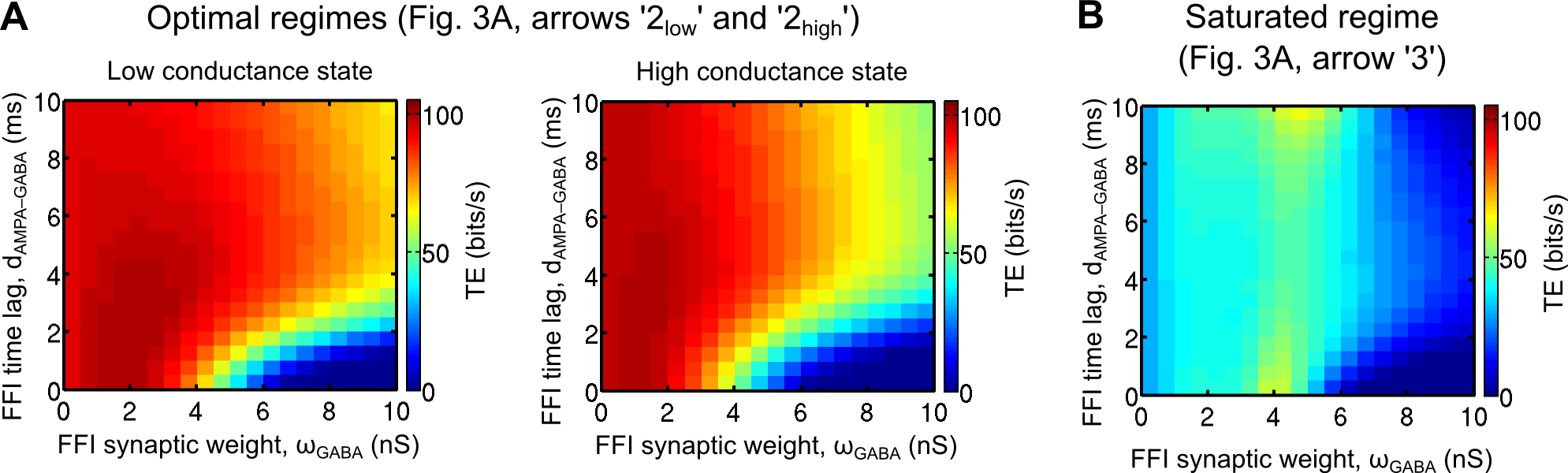

Supplement: Figure S4 — Feedforward inhibition to the cortical cell helps sensory signal transfer in the saturated regime. A. Transfer efficiency as a function of the feedforward inhibition GABAA synaptic weight and time lag (see Methods) for both optimal regimes shown in Figure 3A. B. Similar to A for the saturated regime. (TIF) [file pcbi.1003401.s004.tif]

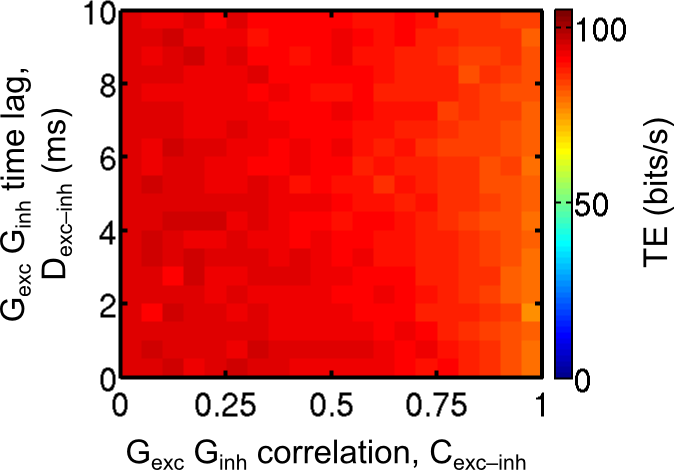

Supplement: Figure S5 — Synaptic bombardment excitation and inhibition interplay in TC model cells. Numerical explorations of the temporal correlations between the excitatory and the inhibitory components of the corticothalamic input at the single cell level. Transfer efficiency is plotted as a function of the excitatory–inhibitory conductance correlation strength and the inhibitory conductance time lag (see Methods). (TIF) [file pcbi.1003401.s005.tif]

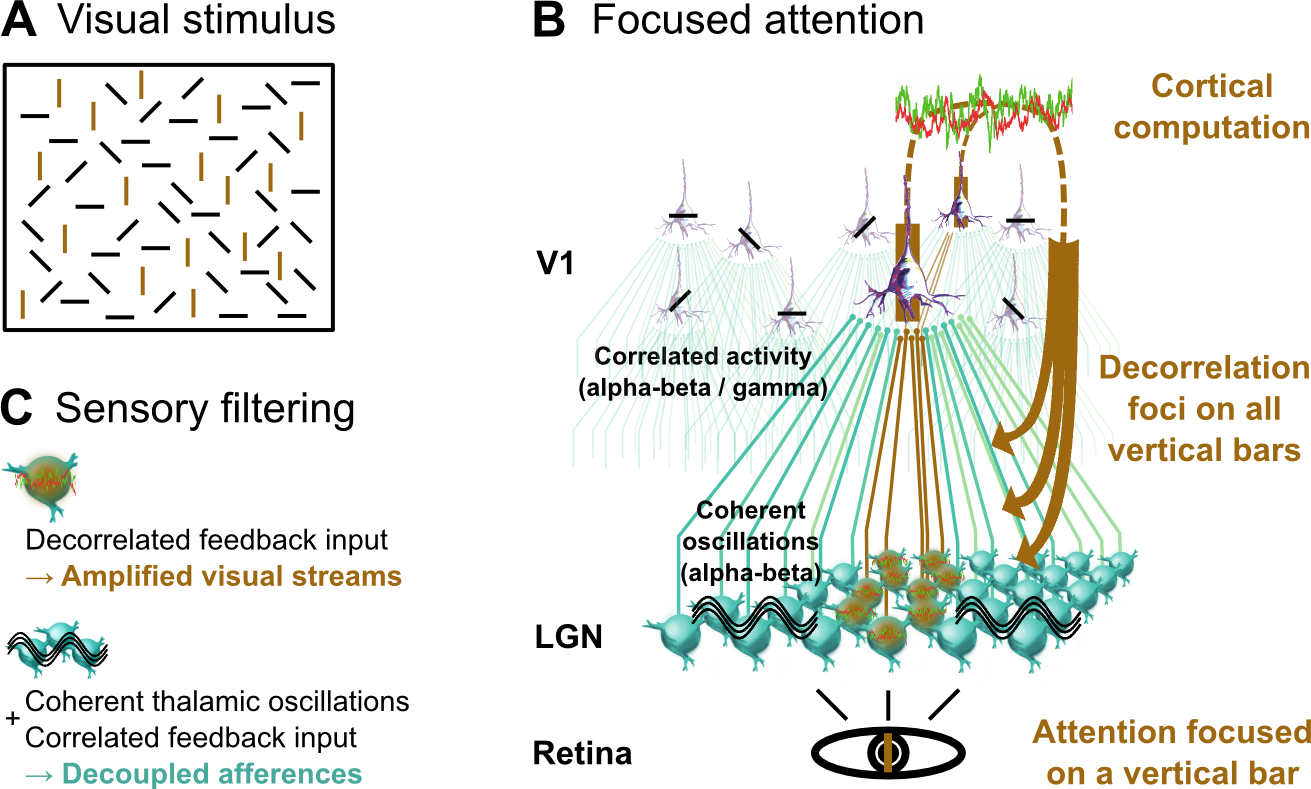

Supplement: Figure S6 — Speculative role of synaptic bombardment decorrelation and thalamic oscillation coherence in focused attention. A. Visual stimulation composed of bars of various orientation. Focusing attention on a single bar (for instance vertical) will slowly segregate all other bars of same orientation from the context made of other bars of dissimilar orientation. Vertical bars are colored in brown for illustration purposes only. B. Presumed functional steps involved when focusing attention on a vertical bar. Vertical bars shown on each neuron illustrate the orientation preference. Columnar organization of V1 circuits is not illustrated although each neuron shown in this schema belong to a different orientation column. An initial decorrelation of activity in cortical area V1 is generated at the retinotopic location of the focused bar. This decorrelated activity is propagated to other regions whose orientation preference match the orientation of the focused bar. A decorrelated corticothalamic feedback is then sent to dLGN target neurons which are specifically tuned to detect features matching a bar of similar orientation. Other thalamic regions that receive no decorrelated feedback would develop synchronized oscillations. More detailed explanations of this hypothesis are provided in Text S1. C. Proposed selective attention mechanisms for sensory signal filtering. Foci of decorrelated corticothalamic activity amplify the visual streams whose features match the bars of vertical orientation while synchronized oscillations in the thalamus reduce the sensory transfer of visual features related to the bars of other orientation. (TIF) [file pcbi.1003401.s006.tif]
