# Supplementary material for: Cortically-Controlled Population Stochastic Facilitation as a Plausible Substrate for Guiding Sensory Transfer across the Thalamic Gateway
Source: PLoS Comput Biol. 2013 Dec 26;9(12):e1003401. doi: 10.1371/journal.pcbi.1003401 (PMC3873227; doi:10.1371/journal.pcbi.1003401)
Supplement: Text S1 — Focused attention: A synaptic bombardment decorrelation hypothesis. Supporting evidences for the implication of synaptic bombardment decorrelation in focused attention. We propose a phenomenological model based on the segregation effect described in Figure S6. Based upon the findings of the present study, we make the prediction of the existence of dynamic functional maps of correlation and decorrelation in V1 and the thalamus. These non-classical maps provide a putative mechanism for the implementation of selective sensory attention in the thalamocortical system. (DOC) [file pcbi.1003401.s007.doc]

**Cortically-controlled population stochastic facilitation as a plausible substrate for guiding sensory transfer across the thalamic gateway**

**Text S1**

**Sébastien Béhuret^1,*^, Charlotte Deleuze^1^, Leonel Gomez^1,2^, Yves Frégnac^1^, Thierry Bal^1^**

^1^Unité de Neurosciences, Information et Complexité (UNIC), CNRS UPR-3293, Gif-sur-Yvette, France

^2^Laboratorio de Neurociencias, Facultad de Ciencias, Universidad de la República Oriental del Uruguay

*Correspondence: [behuret@unic.cnrs-gif.fr](mailto:behuret@unic.cnrs-gif.fr), [bal@](mailto:bal@unic.cnrs-gif.fr)[unic.cnrs-gif.fr](mailto:behuret@unic.cnrs-gif.fr)

**Focused attention: a synaptic bombardment decorrelation hypothesis**

A number of experimental observations indicate that the level of correlation of neuronal activity in the thalamus and cortex changes during focused attention. On the one hand, some studies report increased level of correlation, in particular in the alpha-beta (8–30 Hz) and gamma (30–80 Hz) bands [1–4]. On the other hand, other studies report decreased correlations in LFP signals during attentive tasks [5,6] (see also [3] for alpha-beta decrease). Our hypothesis might reconcile these opposing findings via a unique mechanism: focusing —by decreasing correlations— the appropriate circuitry onto the attended stimulus feature as suggested by [7], but also by imposing increased correlations in the subnetworks involved in the coding of other features, irrelevant to the task. The originality of our proposal is that, for a given attentional task, antagonist effects can be observed: our prediction is that the retinotopic region of the thalamocortical circuitry being the focus of attention will present a low level of correlations, while regions to be neglected outside of the focus will present a high level of correlations. LFP recordings which primarily reflect synaptic activity local coherence pick up signals from restricted cell populations and at this scale the increase or decrease of correlation will entirely depend upon the location of the electrode, like for the classical orientation or ocular dominance maps in V1. We predict the existence of dynamic functional maps for correlation and decorrelation in V1 and in the thalamus. These maps differ from the classical maps cited above in that they should vary according to the stimuli and the attentional context, and their distribution should vary from moment to moment.

We illustrate this concept with a visual stimulus composed of bars of random orientation chosen among four with a 45° angle difference (Fig. S6A). When we focus our attention on a single bar (for instance vertical), then all other bars of same orientation become segregated from the context made of other bars of dissimilar orientation. This effect is rather slow and demands an attentional effort, unlike the classical pop-out that is automatic and fast [8]. This slower process could depend on a feedback from high-level cortical areas [8], and on horizontal synaptic connections in V1. Note that the color of the bar is not an issue here, the effect is the same when bars are all of the same color.

We describe a phenomenological model for this visual effect in several steps illustrated in Figure S6B:

**1** – **Initial decorrelation of activity in cortical area V1:** In the retinotopic stream activated by the attended vertical bar, retinal ganglion cells discharge in synchrony due to the spatiotemporal coincidence of the stimulus. In the dLGN, thalamocortical relay cells are activated by the synchronous retinal inputs and in turn they activate their target cortical cell of same orientation selectivity in V1. These synchronous discharges can generate decorrelation in the cortex via mechanisms described in [9,10]. A likely source of decorrelation could be due to the attentional feedback from high-level visual areas [8]. Outside of the focused area, correlated activities are essentially generated by an intracortical recurrent activity and horizontal activity. The increased synchronization of cortical networks in the gamma frequency may stem from elevated oscillatory retinal frequencies [11], intracortical mechanisms including high-frequency bursting [12] and interneurons activity [13]. Similarly, increased synchronization in the alpha-beta frequency range may stem from cortex and intrinsic oscillatory activities in the thalamus (see below). In contrast, within the target region of the focused attention, we predict that a LFP electrode precisely located within the tiny focused area in V1 would reveal decreased correlation levels in the alpha-beta and/or gamma frequency ranges.

**2** – **Propagating waves of decorrelation in V1:** The initial decorrelation propagates to other columns of vertical orientation via connections between columns of same orientation [14–16]. In this situation, an LFP electrode placed in one such column of same orientation in V1 would detect a decreased level of of alpha-beta and/or gamma activity correlation. In contrast the correlation would be higher in other columns of different orientation preference, an effect which could be further amplified by the lack of thalamocortical input resulting from the filtering of the sensory information. This notion of sensory filtering is explicated below.

**3** – **Foci of corticothalamic decorrelation:** Areas of decorrelated activity in V1 send a decorrelated corticothalamic synaptic activity to their target neurons in the dLGN and in the NRT. We infer from the retinotopic organization of the top-down projections that the target neurons in the dLGN are specifically tuned to detect features matching a bar similar to the one being focused. In all other target areas of the thalamus corresponding to the feedback of cortical columns of different orientation selectivity, the global activity pattern of the descending cortical input remains correlated, and imposes a reduction in the efficiency of the sensory information transfer.

**4** – **Oscillatory activity in the thalamus:** Oscillations in the thalamus are generated by various known mechanisms such as intrinsic properties, gap junctions and synaptic interactions with NRT [17,18]. Oscillations in the alpha-beta range are synchronized in V1 and dLGN during attention [4], an effect which may rely on corticothalamic projections [19]. Therefore, outside of the corticothalamic foci of decorrelation, thalamic oscillations in the alpha-beta range might be initiated or reinforced by the synchronizing action of the descending inputs. In turn, thalamic oscillations might further contribute to the correlation of the cortical activity in the alpha-beta and/or gamma range. According to our hypothesis, such a correlated activity carried within the thalamocortical loop would result in a filtering of the sensory information which is unrelated to the focused vertical bar or other bars of same orientation.

As we have shown in this paper, the occurrence of synchronized oscillations in the thalamus reduces sensory transfer (see Fig. 7C; [20]) while decorrelated cortical input boosts sensory transfer (see Fig. 4E,F and 5B,D).

In summary, we envision a mechanism of selective sensory attention by which top-down cortical inputs could create an ever changing landscape of islands of highly efficient sensory spike transfer in a network otherwise functionally decoupled from its inputs (Fig. S6C).

**References**

1. Bekisz M, Wróbel A (1993) 20 Hz rhythm of activity in visual system of perceiving cat. Acta neurobiologiae experimentalis 53: 175–182. Available: http://www.ncbi.nlm.nih.gov/pubmed/8317245. Accessed 13 June 2013.

2. Bouyer JJ, Montaron MF, Rougeul A (1981) Fast fronto-parietal rhythms during combined focused attentive behaviour and immobility in cat: cortical and thalamic localizations. Electroencephalography and clinical neurophysiology 51: 244–252.

3. Fries P, Reynolds JH, Rorie AE, Desimone R (2001) Modulation of oscillatory neuronal synchronization by selective visual attention. Science (New York, NY) 291: 1560–1563. Available: http://www.ncbi.nlm.nih.gov/pubmed/11222864. Accessed 13 June 2013.

4. Bekisz M, Wróbel A (2003) Attention-dependent coupling between beta activities recorded in the cat’s thalamic and cortical representations of the central visual field. European Journal of Neuroscience 17: 421–426. doi:10.1046/j.1460-9568.2003.02454.x.

5. Cohen MR, Maunsell JHR (2009) Attention improves performance primarily by reducing interneuronal correlations. Nature neuroscience 12: 1594–1600. doi:10.1038/nn.2439.

6. Mitchell JF, Sundberg KA, Reynolds JH (2009) Spatial attention decorrelates intrinsic activity fluctuations in macaque area V4. Neuron 63: 879–888. doi:10.1016/j.neuron.2009.09.013.

7. Sillito AM, Jones HE, Gerstein GL, West DC (1994) Feature-linked synchronization of thalamic relay cell firing induced by feedback from the visual cortex. Nature 369: 479–482. Available: http://www.ncbi.nlm.nih.gov/pubmed/8202137. Accessed 13 June 2013.

8. Hochstein S, Ahissar M (2002) View from the top: hierarchies and reverse hierarchies in the visual system. Neuron 36: 791–804. Available: http://www.ncbi.nlm.nih.gov/pubmed/12467584. Accessed 27 May 2013.

9. Renart A, De la Rocha J, Bartho P, Hollender L, Parga N, et al. (2010) The asynchronous state in cortical circuits. Science (New York, NY) 327: 587–590. doi:10.1126/science.1179850.

10. Middleton JW, Omar C, Doiron B, Simons DJ (2012) Neural correlation is stimulus modulated by feedforward inhibitory circuitry. The Journal of neuroscience : the official journal of the Society for Neuroscience 32: 506–518. Available: http://www.pubmedcentral.nih.gov/articlerender.fcgi?artid=3282531&tool=pmcentrez&rendertype=abstract. Accessed 23 May 2013.

11. Troy JB, Robson JG (2009) Steady discharges of X and Y retinal ganglion cells of cat under photopic illuminance. Visual Neuroscience 9: 535. doi:10.1017/S0952523800001784.

12. Gray CM, McCormick DA (1996) Chattering cells: superficial pyramidal neurons contributing to the generation of synchronous oscillations in the visual cortex. Science (New York, NY) 274: 109–113.

13. Traub RD, Spruston N, Soltesz I, Konnerth A, Whittington MA, et al. (1998) Gamma-frequency oscillations: a neuronal population phenomenon, regulated by synaptic and intrinsic cellular processes, and inducing synaptic plasticity. Progress in neurobiology 55: 563–575.

14. Bosking WH, Zhang Y, Schofield B, Fitzpatrick D (1997) Orientation selectivity and the arrangement of horizontal connections in tree shrew striate cortex. The Journal of neuroscience : the official journal of the Society for Neuroscience 17: 2112–2127.

15. Angelucci A, Levitt JB, Walton EJS, Hupe J-M, Bullier J, et al. (2002) Circuits for local and global signal integration in primary visual cortex. The Journal of neuroscience : the official journal of the Society for Neuroscience 22: 8633–8646.

16. Lund JS, Angelucci A, Bressloff PC (2003) Anatomical substrates for functional columns in macaque monkey primary visual cortex. Cerebral cortex (New York, NY : 1991) 13: 15–24.

17. Hughes SW, Lörincz M, Cope DW, Blethyn KL, Kékesi K a, et al. (2004) Synchronized Oscillations at α and θ Frequencies in the Lateral Geniculate Nucleus. Neuron 42: 253–268. doi:10.1016/S0896-6273(04)00191-6.

18. McCormick DA, Bal T (1997) Sleep and arousal: thalamocortical mechanisms. Annual review of neuroscience 20: 185–215. doi:10.1146/annurev.neuro.20.1.185.

19. Contreras D, Destexhe A, Sejnowski TJ, Steriade M (1996) Control of Spatiotemporal Coherence of a Thalamic Oscillation by Corticothalamic Feedback. Science 274: 771–774. doi:10.1126/science.274.5288.771.

20. Le Masson G, Renaud-Le Masson S, Debay D, Bal T (2002) Feedback inhibition controls spike transfer in hybrid thalamic circuits. Nature 417: 854–858. doi:10.1038/nature00825.
